# Supplementary material for: The evolution of ovary-biased gene expression in Hawaiian Drosophila
Source: PLoS Genet. 2023 Jan 23;19(1):e1010607. doi: 10.1371/journal.pgen.1010607 (PMC9894553; doi:10.1371/journal.pgen.1010607)
Supplement: S1 Text — Additional figures and text describing analysis methods, as well as tables of sequencing statistics and results. (PDF) [file pgen.1010607.s001.pdf]

The evolution of ovary-biased gene expression in Hawaiian *Drosophila* - Supplementary methods and tables

Samuel H. Church[1,2,*] Catriona Munro[3] Casey W. Dunn[2]
Cassandra G. Extavour[1,4,5]

Contents

1 Supplementary figures **2**

1.1 Background 2

1.2 Analysis pipeline 3

1.3 Differential gene expression 4

1.4 Linear modeling 10

1.5 Phylogenetic analysis 13

2 Tables **15**

2.1 Collection and sequencing 15

2.2 Core genes 21

References 24

[1] Department of Organismic and Evolutionary Biology, Harvard University, Cambridge, MA 02138, USA

[2] Current address: Department of Ecology and Evolutionary Biology, Yale University, New Haven, CT 06520, USA

[3] Collège de France, PSL Research University, CNRS, Inserm, Center for Interdisciplinary Research in Biology, 75005 Paris, France

[4] Department of Molecular and Cellular Biology, Harvard University, Cambridge, MA 02138, USA

[5] Howard Hughes Medical Institute, Chevy Chase, MD 20815

* corresponding author: samuelhchurch@gmail.com

1 Supplementary figures

1.1 Background

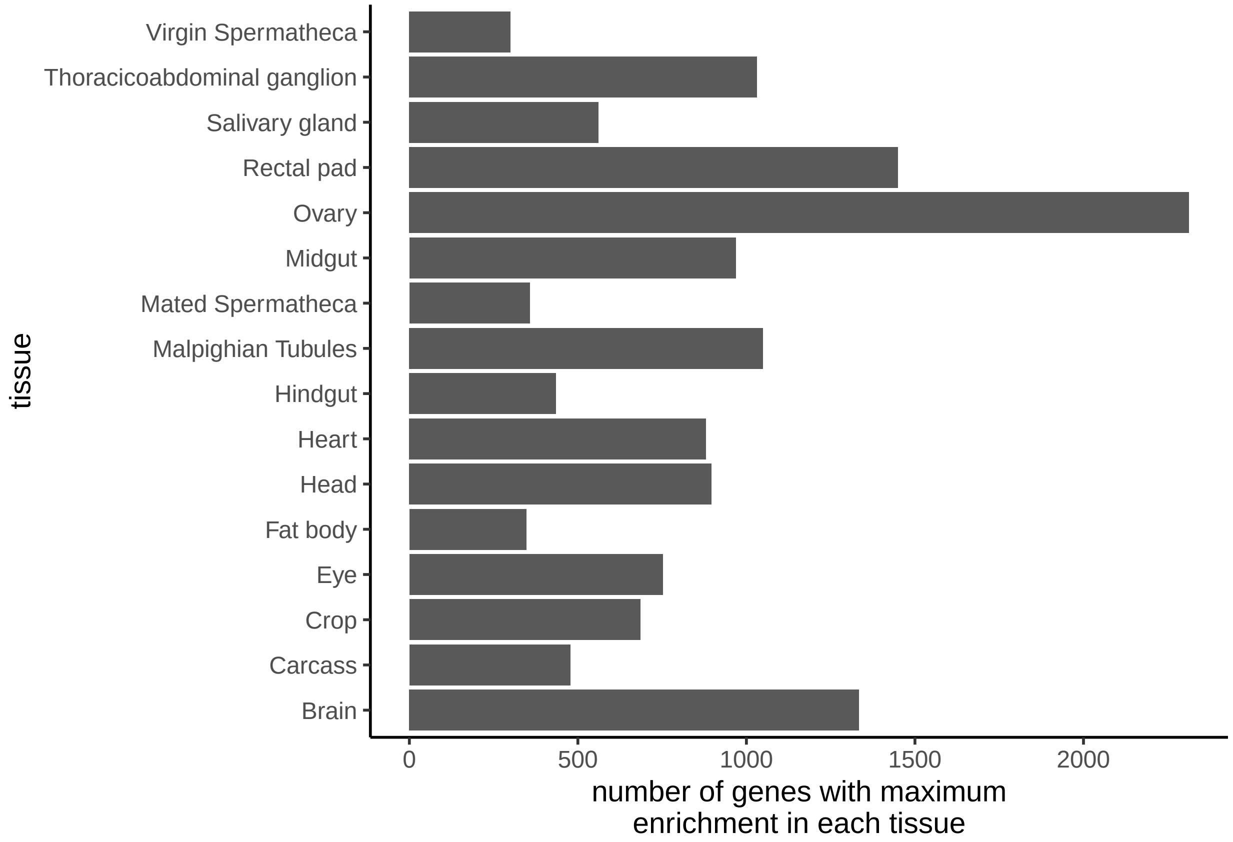

Figure A: **Comparing across *Drosophila melanogaster* female tissues in the FlyAtlas2 dataset[1], more genes show highest enrichment in the ovary than any other tissue.**

1.2 Analysis pipeline

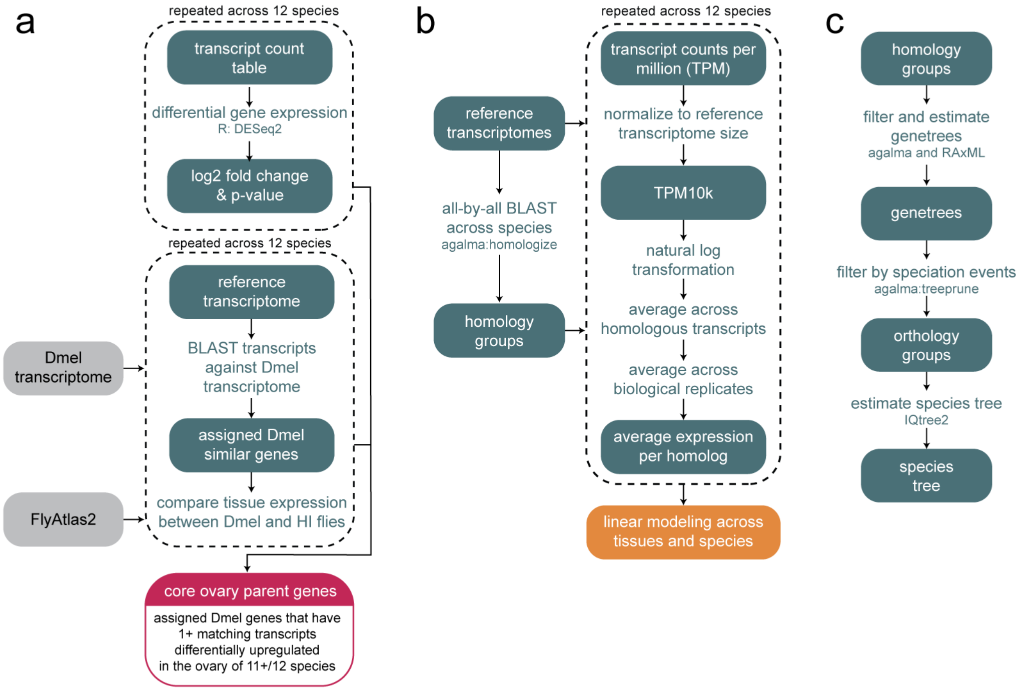

Figure B: **Analysis pipeline for (a) identifying core ovary genes, (b) linear modeling of expression, and (c) estimating the species level phylogeny. Dmel = *Drosophila melanogster*. HI flies = Hawaiian Drosophilidae.**

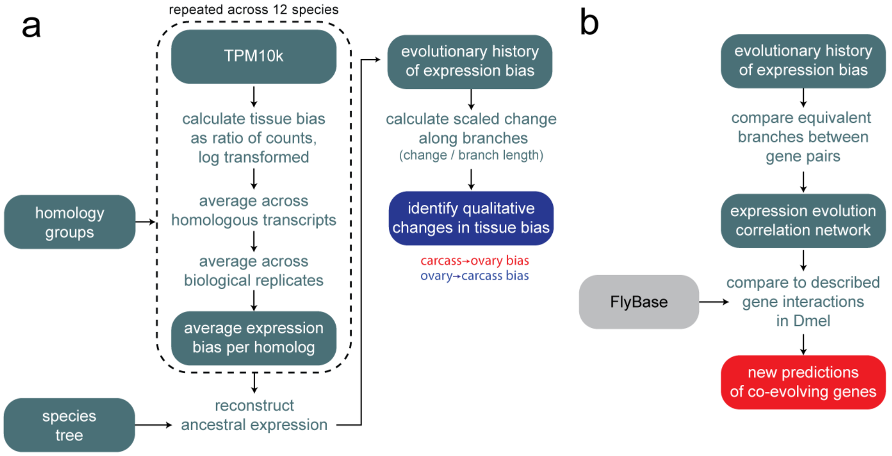

Figure C: **Analysis pipeline for (a) identifying evolutionary changes in expression bias, and (b) estimating correlation of expression evolution between genes.**

1.3 Differential gene expression

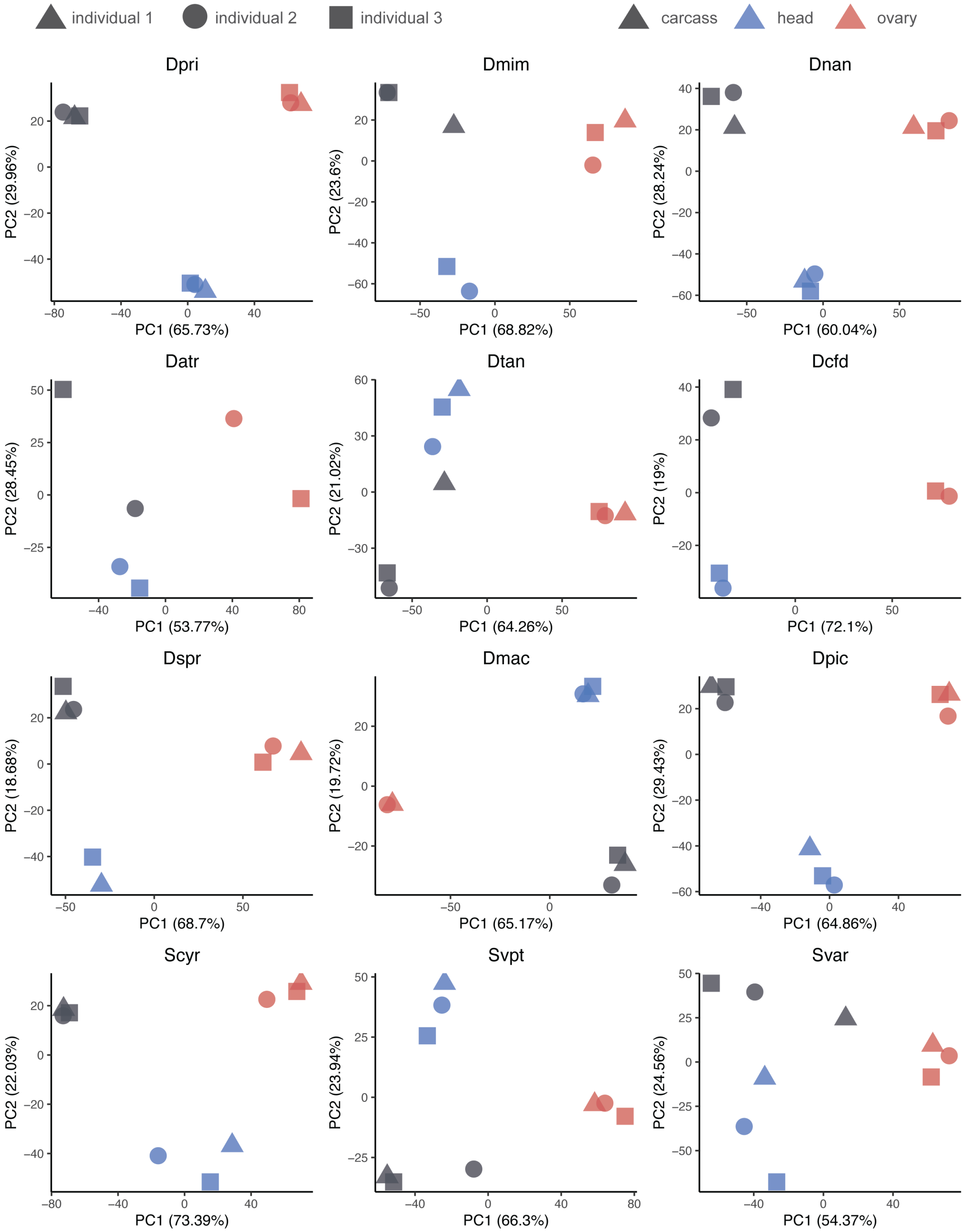

Figure D: **Principal component analysis within species.** Species abbreviations correspond to names shown in Fig 1B.

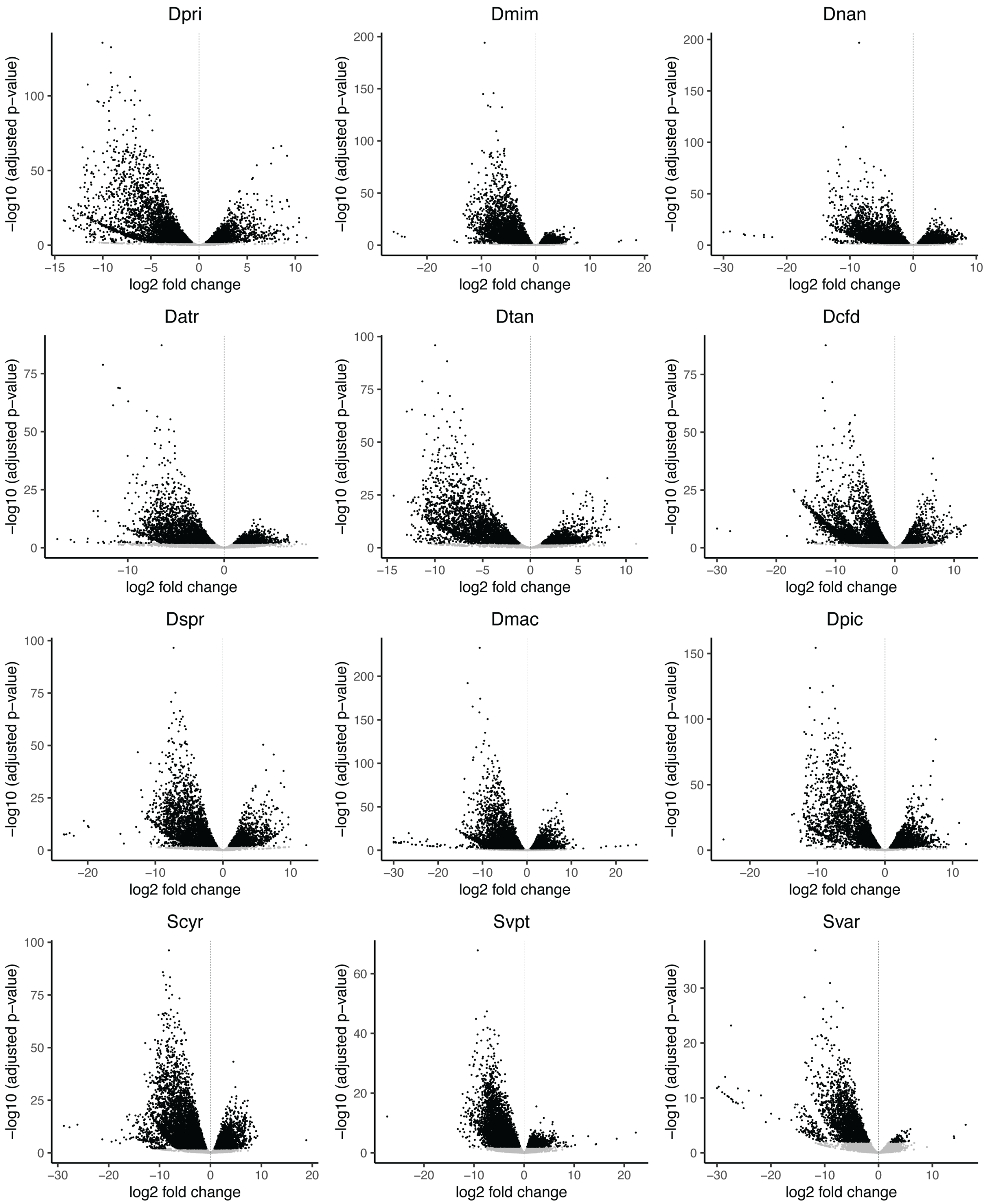

Figure E: **Differential gene expression volcanos plot comparing ovary and carcass across species.** Species abbreviations correspond to names shown in Fig 1B. Positive fold changes (points on the right hand side of the volcano) indicate higher expression in the ovary than in the carcass.

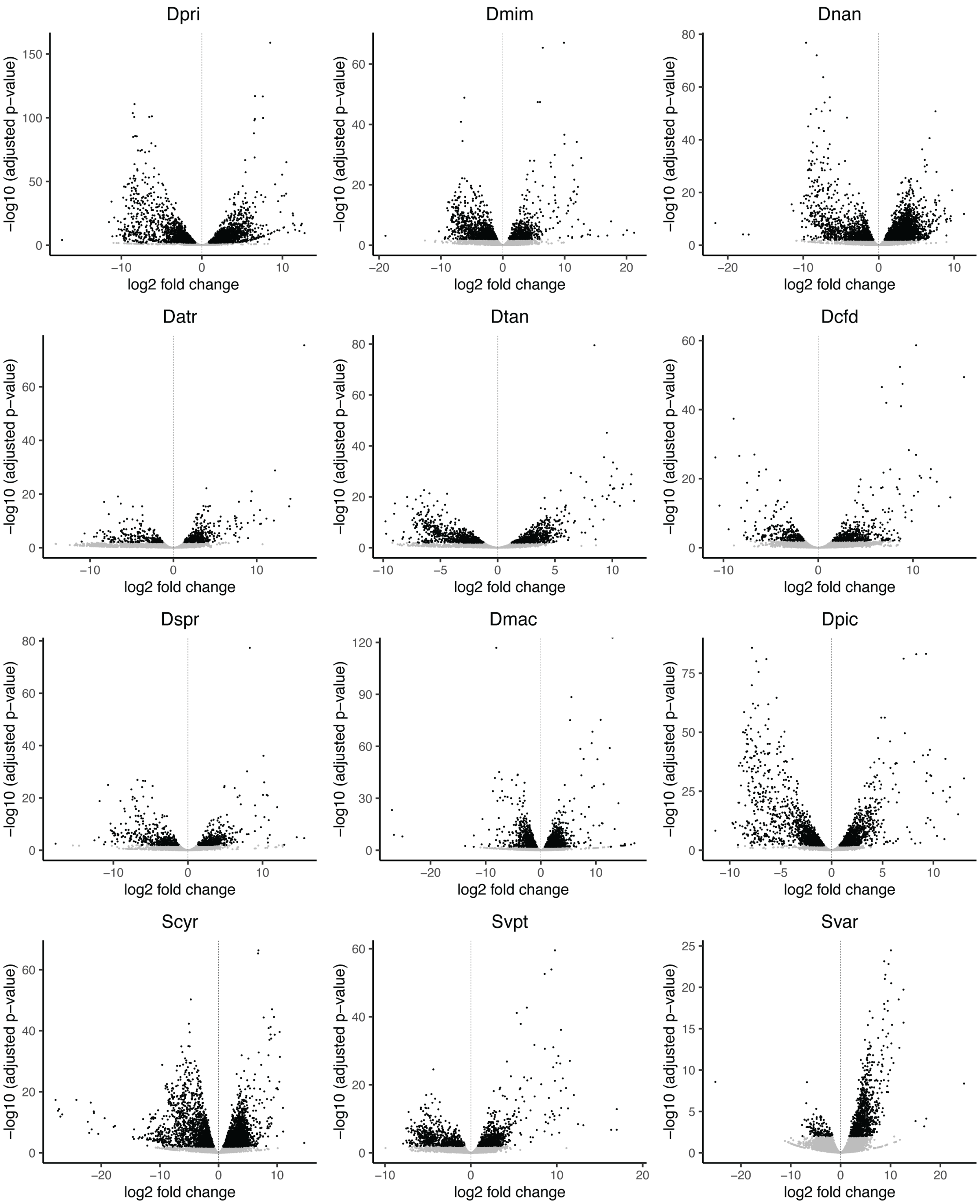

Figure F: **Differential gene expression volcanos plot comparing head and carcass across species.** Species abbreviations correspond to names shown in Fig 1B. Positive fold changes (points on the right hand side of the volcano) indicate higher expression in the head than in the carcass.

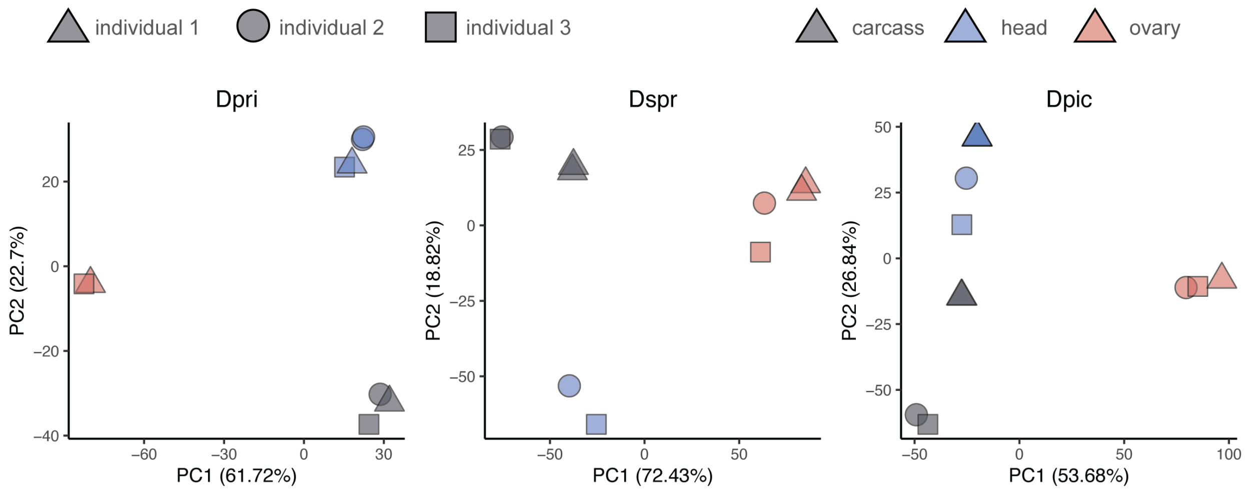

Figure G: **Principal component analysis showing resequenced libraries.** Species abbreviations correspond to names shown in Fig 1B.

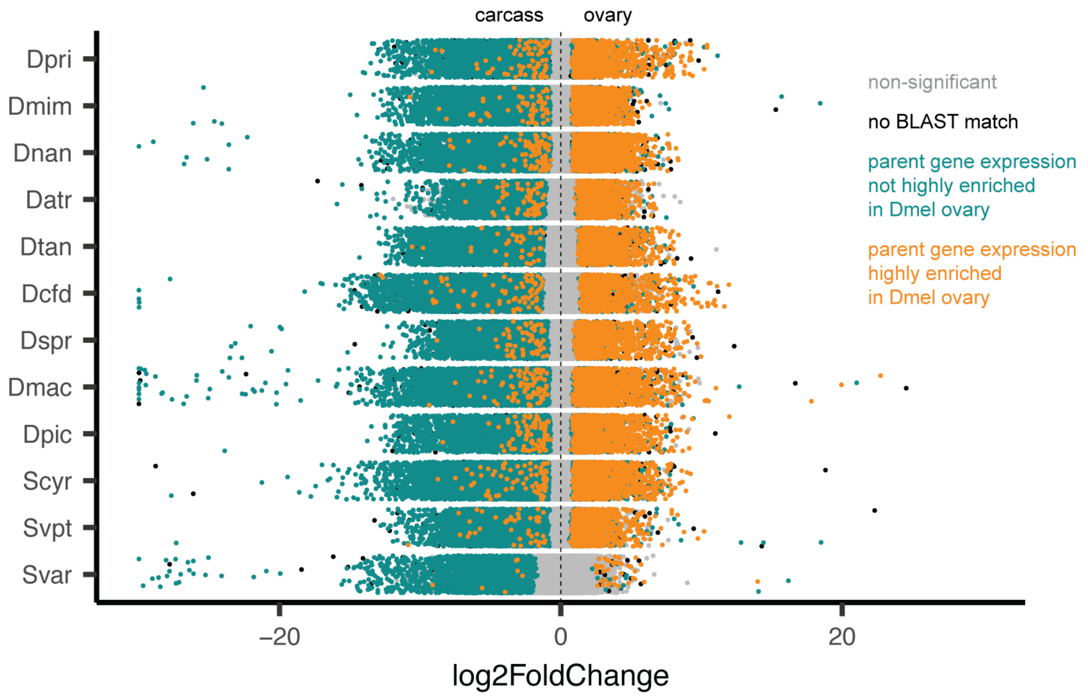

Figure H: **Differential gene expression analysis across species, colored by *D. melanogaster* expression enrichment.** Points in orange indicate transcripts that match *D. melanogaster* genes that are highly enriched in the ovary, according to FlyAtlas2[1]. Cyan are transcripts matching genes that are not highly enriched in the ovary. Black are transcripts without a BLAST match in *D. melanogaster*. Gray are non-significantly differentially expressed transcripts.

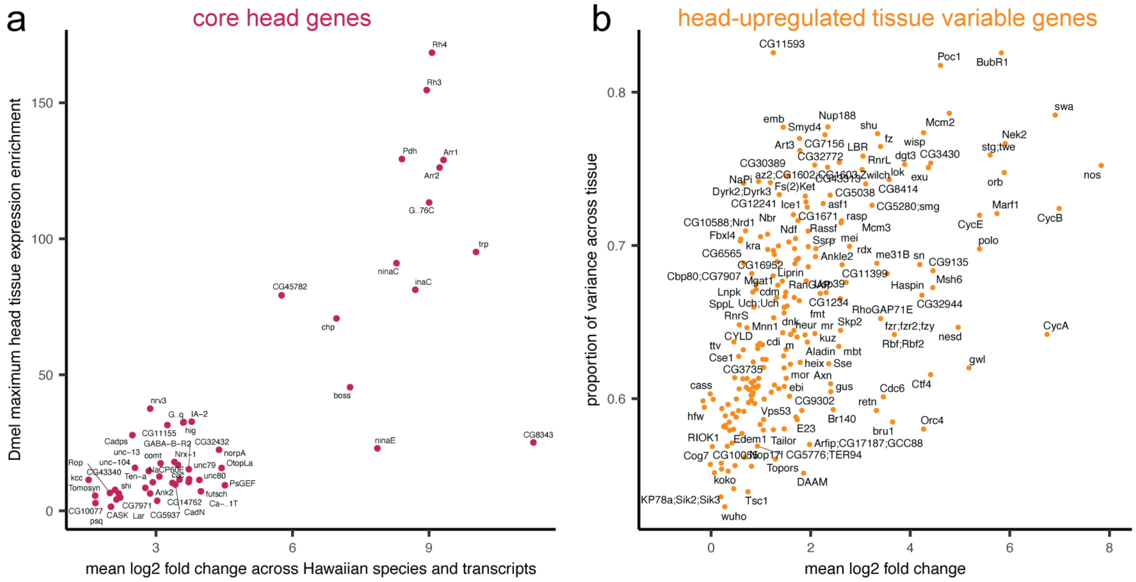

Figure I: **Core head genes.** a, Core head genes, plotted by mean expression change across Hawaiian species to the maximum head, brain, or eye enrichment values from *D. melanogaster*, as reported in FlyAtlas2[1]. Core genes are annotated with the gene symbol from *D. melanogaster*. b, Tissue-variable genes identified in a linear model analysis comparing head and carcass tissues, showing head-upregulated TVGs. Genes are annotated with the gene symbol from the *D. melanogaster* sequences in the same homology group.

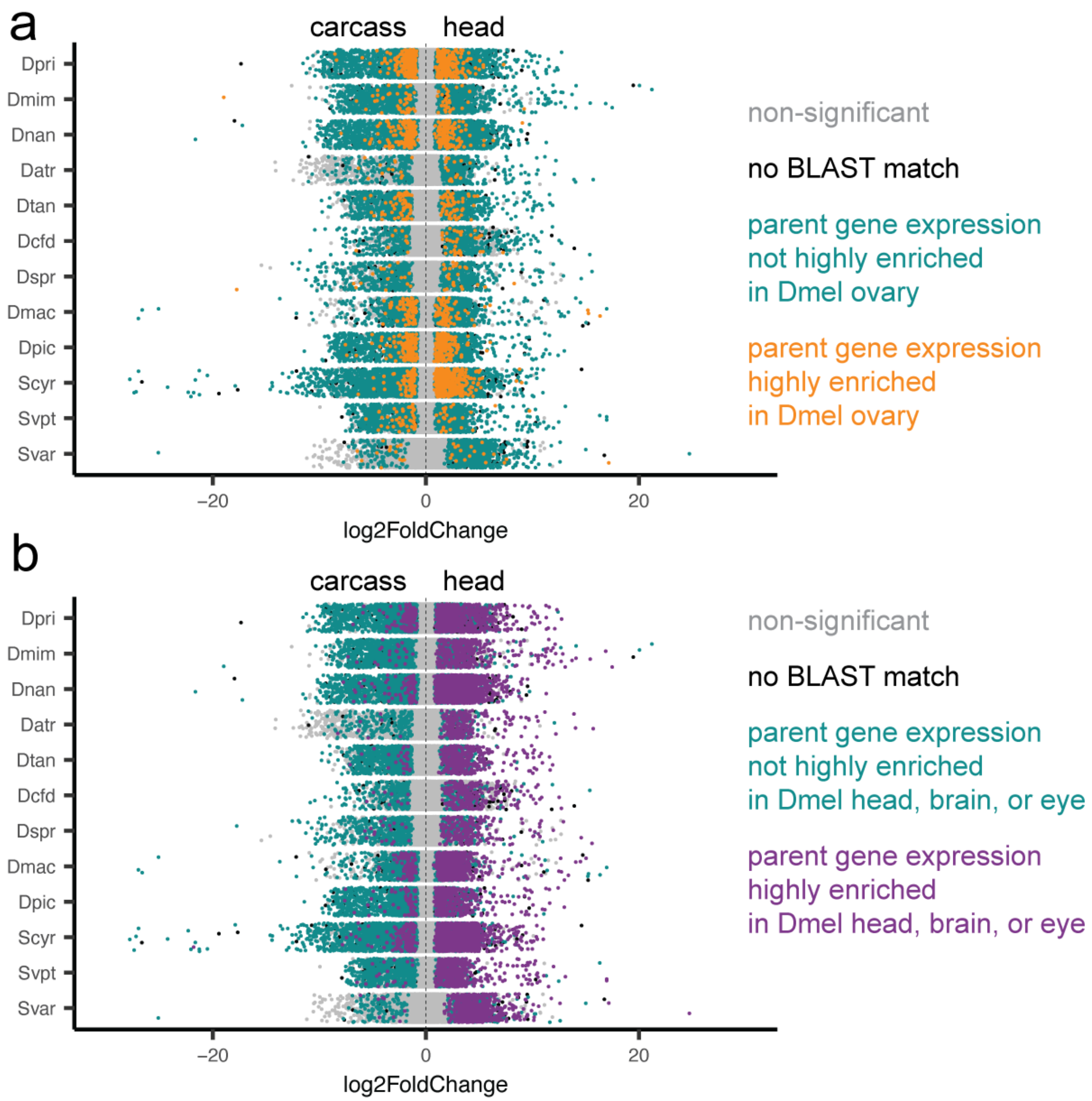

Figure J: **Differential gene expression analyses comparing head and carcass, colored by *D. melanogaster* expression enrichment.** a, Points in orange indicate transcripts that match *D. melanogaster* genes that are highly enriched in the ovary, according to FlyAtlas2[1]. Cyan are transcripts matching genes that are not highly enriched in the ovary. Black are transcripts without a BLAST match in *D. melanogaster*. Gray are non-significantly differentially expressed transcripts. b, The same plot, with purple points as transcripts that are highly enriched in the head, brain, or eye of *D. melanogaster*, according to FlyAtlas2[1].

1.4 Linear modeling

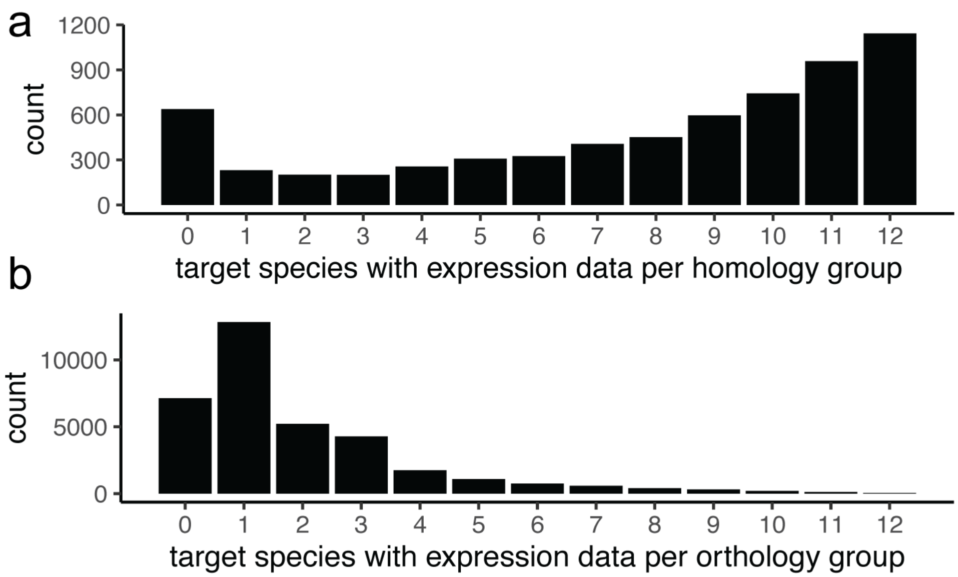

Figure K: **Representation across homology and orthology groups.** a, The number of target species with expression data per homology group, inferred with the agalma pipeline using an all-by-all BLAST approach between the twelve reference transcriptomes and twelve other published Drosophilidae genomes. Groups with zero species are those that contain only genes from the species with published genomes, and not the twelve target species sequenced this study. b, The number of target species with expression data per orthology group, inferred with the agalma pipeline, using gene trees to identify orthologs.

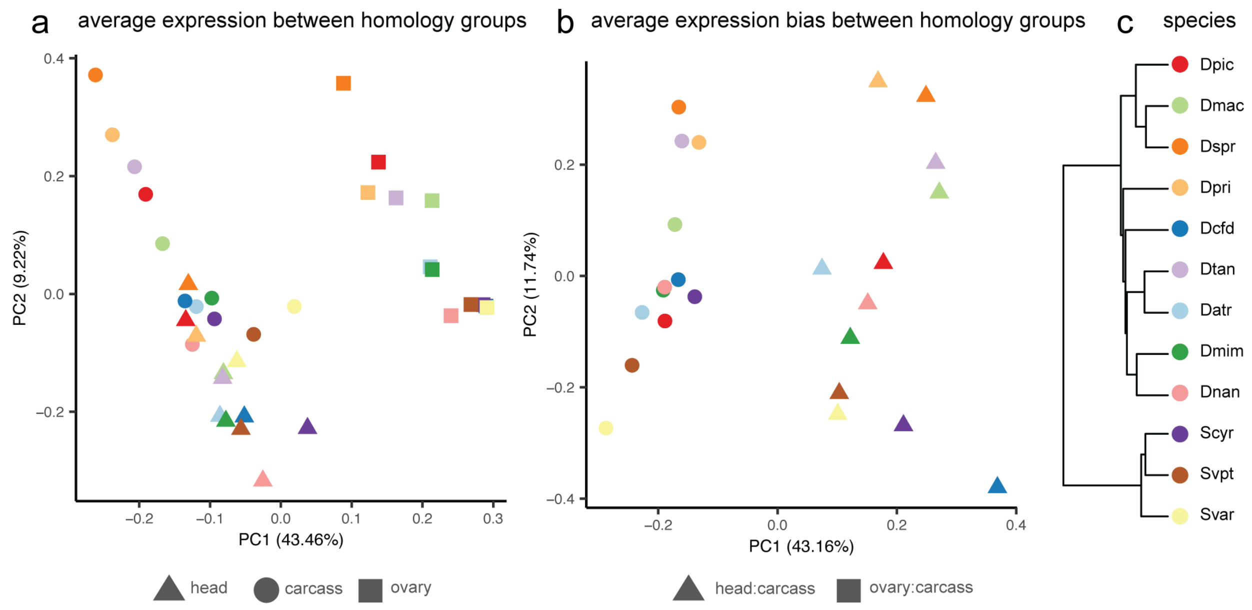

Figure L: **Principal component analysis across species.** a, PCA using average expression across genes grouped by homology, as inferred with the agalma pipeline. b, PCA using average expression bias, calculated as an expression ratio between the ovary and carcass or head and carcass. Ratios are calculated across genes grouped by homology. c, Phylogeny of species shown in a and b.

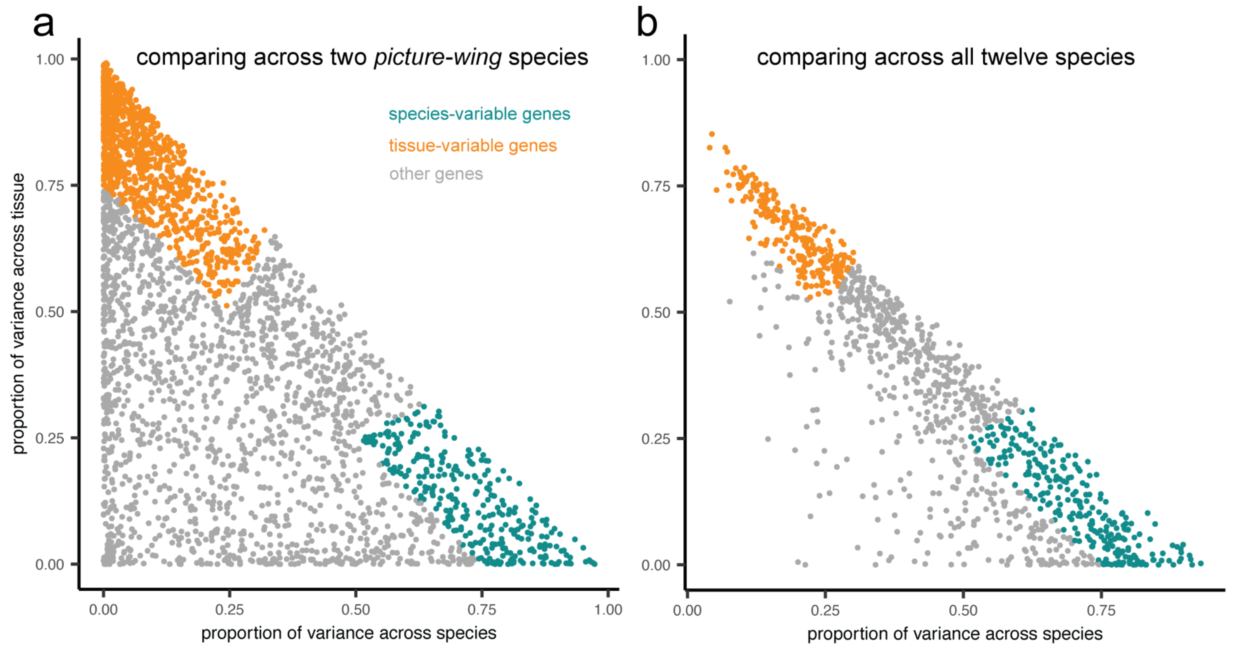

Figure M: **Linear model results by gene.** a, Results of a linear model analysis between genes, grouped by homology as inferred in the agalma pipeline, comparing two *picture-wing* species, *D. macrothrix* and *D. sproati*. Orange points are tissue-variable genes (TVGs), cyan are species-variable genes (SVGs), gray are neither. b, Results of the same analysis across all twelve Hawaiian drosophilid species studied here.

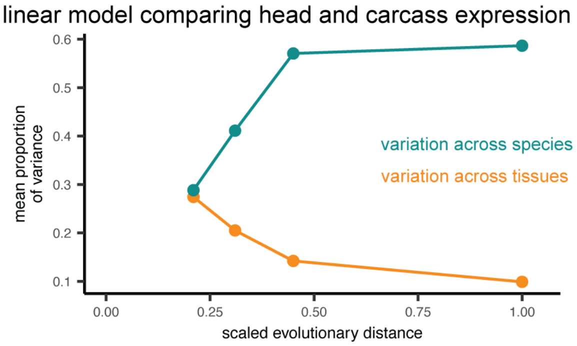

Figure N: **Proportion of variance across species and tissues, comparing head and carcass.** The mean proportion of variance across genes attributed to species and tissues, comparing the head and carcass across four clades. Clades correspond to those shown in Fig 3A.

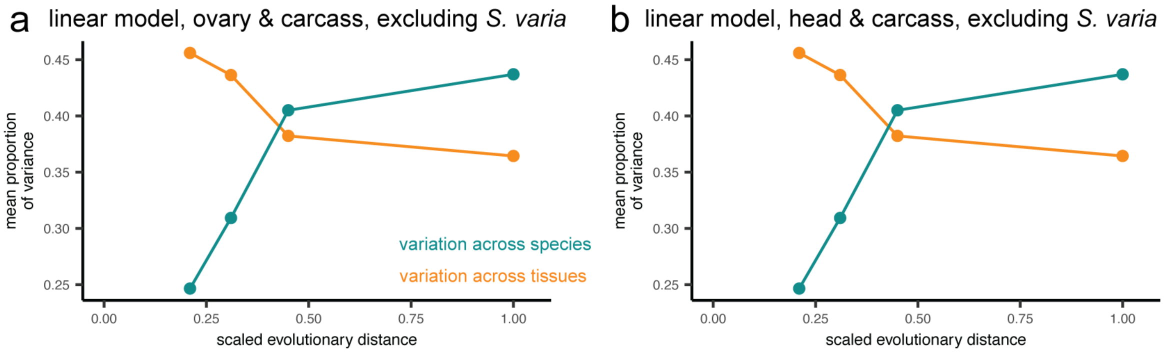

Figure O: **Proportion of variance across species and tissues, excluding *S. varia*.** a, The mean proportion of variance across genes attributed to species and tissues, comparing the ovary and carcass across four clades, excluding *S. varia* from clade D. b, The same plot, comparing the head and carcass. Clades correspond to those shown in Fig 3A.

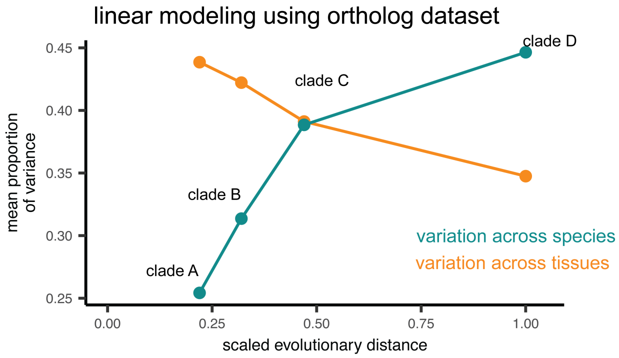

Figure P: **Proportion of variance across species and tissues, calculated using a dataset of 1:1 orthologs.** The mean proportion of variance across genes attributed to species and tissues, comparing the ovary and carcass across four clades. Sample sizes are for clade A: 2,185 orthology groups, clade B: 918, clade C: 87, and clade D: 37.

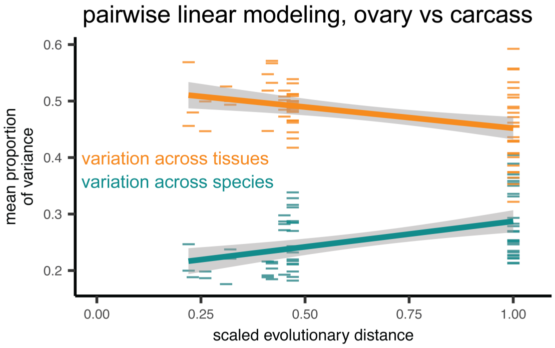

Figure Q: **Proportion of variance across species and tissues, calculated using pairwise combinations of species.** The mean proportion of variance across genes attributed to species and tissues, comparing the ovary and carcass across all pairwise combinations of twelve species, shown as colored bars. Lines and gray shadow shows the regression and 95% confidence interval using a linear model.

1.5 Phylogenetic analysis

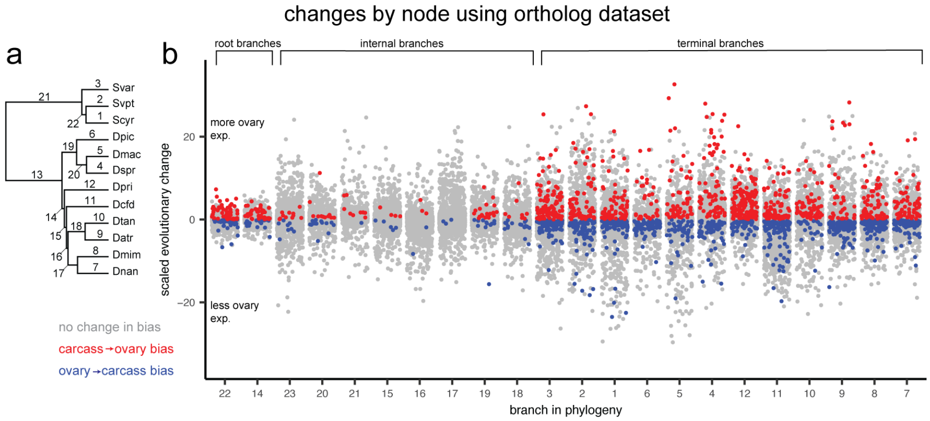

Figure R: **Changes in ovary-biased expression in strict orthologs.** a, The phylogeny with all 22 branches numbered. b, The distribution of changes in expression of orthologs, grouped by branch, with random jitter on the x-axis within each group. Points colored according to the qualitative change in bias, either from more expression in ovary than carcass to less (blue), the reverse (red), or no change in overall bias (gray).

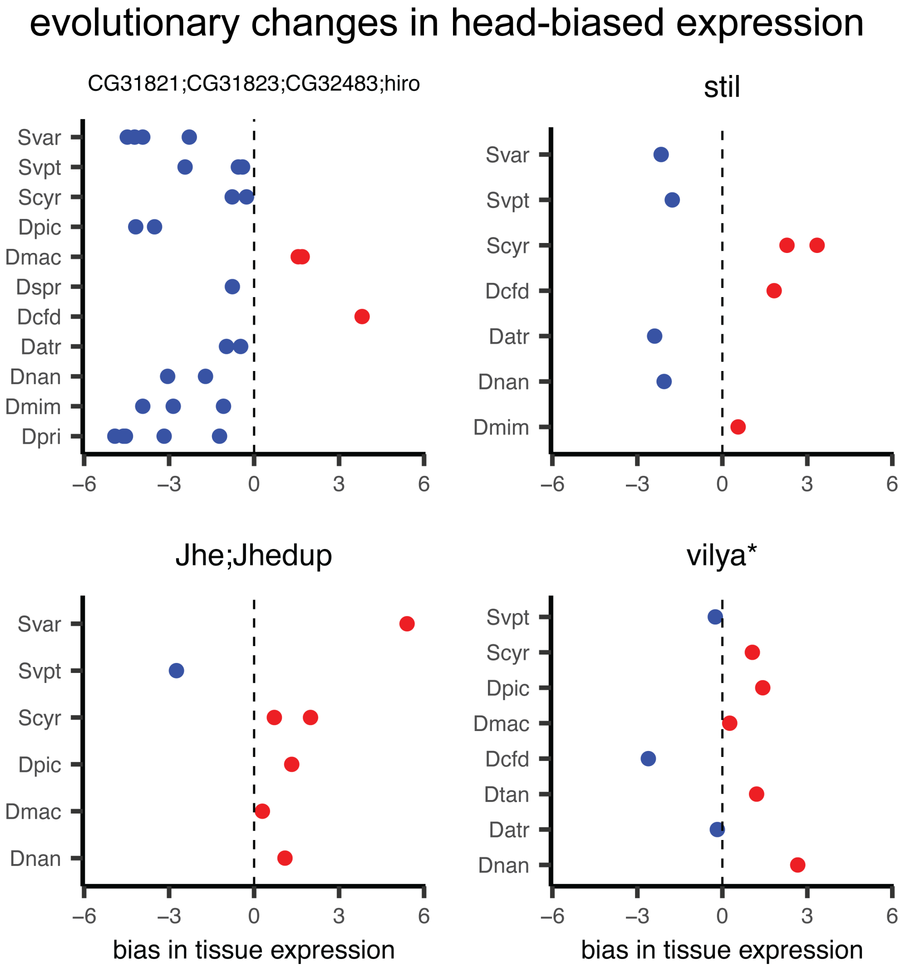

Figure S: **Changes in head-biased expression evolution.** Four genes displaying large swings in relative head expression, showing the expression bias for each transcript colored according to more expression in the head (red) or carcass (blue). Panels are annotated with the gene symbol from the *D. melanogaster* sequences in the same homology group, with the exception of *vilya**, which was annotated using a direct BLAST search since no *D. melanogaster* sequence was present in that group.

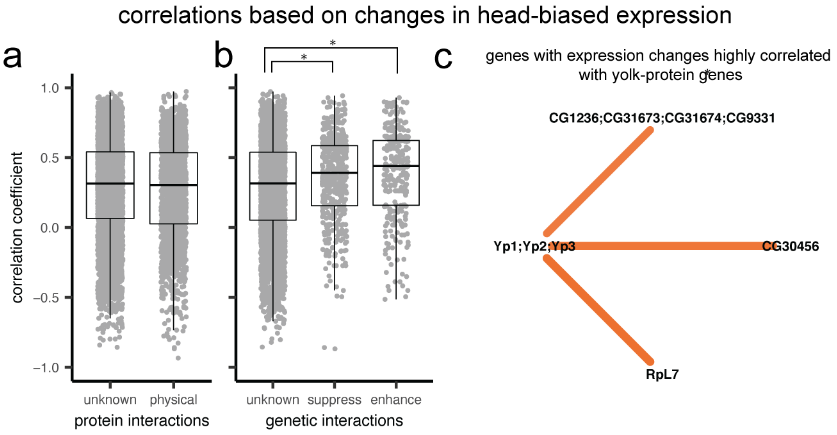

Figure T: **Evolutionary correlations based on head-biased expression evolution.** a-b Comparison of the dsitribution of Pearson's correlation coefficients based on head-biased expression evolution between genes. Asterisks indicate a significant t-test comparison. Box plots indicate mean, upper and lower quartiles, and 1.5x interquartile ranges. a, Genes with no or unknown protein-protein interactions compared to those with reported interactions in FlyBase[2] (maximum p-value=0.256 over 100 replicates). b, Correlation comparison between genes with no or uknown genetic interactions and those reported to have enhancement or suppression interactions in FlyBase (unknown vs. enhancemenet max. p-value=<0.001; unknown vs. suppression max. p-value=<0.001; enhancement vs. suppression p-value=0.248). c, The network of strong correlation partners (absolute correlation >0.825) with the yolk-protein genes. Nodes are annotated with the gene symbols from the *D. melanogaster* sequences from that homology group.

2 Tables

2.1 Collection and sequencing

Table A: Field collection information for sequenced specimens.

individual	species	general site	locality	collection method	permit	collection date	GPS
088C	*Scaptomya cyrtandrae*	Stainback Highway	Army road west	on Cyrtandra platyphylla	DOFAW I1012; NARS special use; Hawai'i island forest reserve access permit	5/29/2017	N19° 33.615' W155° 15.010'
088A	*Scaptomya cyrtandrae*	Stainback Highway	Army road west	on Cyrtandra platyphylla	DOFAW I1012; NARS special use; Hawai'i island forest reserve access permit	5/29/2017	N19° 33.615' W155° 15.010'
088B	*Scaptomya cyrtandrae*	Stainback Highway	Army road west	on Cyrtandra platyphylla	DOFAW I1012; NARS special use; Hawai'i island forest reserve access permit	5/29/2017	N19° 33.615' W155° 15.010'
025A	*Drosophila picticornis*	Koke'e State Park	Awa'awapuhi trail	baits	DOFAW I1012; Koke'e state park K2017-2015; I1012; NARS special use; Kaua'i island forest reserves KPI-2017-114	4/14/2017	N22° 08.481' W159° 38.926'
16.2-1	*Drosophila cf dives*	Hawai'i Volcanoes National Park	Bird park	baits	DOFAW I1012; HAVO-2017-SCI-0017	5/10/2016	N19° 26.3512' W155° 18.2225'
16.1-1	*Drosophila cf dives*	Hawai'i Volcanoes National Park	Bird park	baits	DOFAW I1012; HAVO-2017-SCI-0017	5/10/2016	N19° 26.3512' W155° 18.2225'

Table A: Field collection information for sequenced specimens. *(continued)*

individual	species	general site	locality	collection method	permit	collection date	GPS
18.0-3	*Drosophila mimica*	Hawai'i Volcanoes National Park	Bird park	sweeping Sapindus saponaria leaves	DOFAW I1012; HAVO-2017-SCI-0017	5/10/2016	N19° 26.3512' W155° 18.2225'
040B	*Drosophila mimica*	Hawai'i Volcanoes National Park	Bird park	sweeping Sapindus saponaria leaves	DOFAW I1012; HAVO-2017-SCI-0017	4/17/2017	N19° 26.3512' W155° 18.2225'
040D	*Drosophila mimica*	Hawai'i Volcanoes National Park	Bird park	sweeping Sapindus saponaria leaves	DOFAW I1012; HAVO-2017-SCI-0017	4/17/2017	N19° 26.3512' W155° 18.2225'
040C	*Drosophila mimica*	Hawai'i Volcanoes National Park	Bird park	sweeping Sapindus saponaria leaves	DOFAW I1012; HAVO-2017-SCI-0017	4/17/2017	N19° 26.3512' W155° 18.2225'
032B	*Drosophila nanella*	Koke'e State Park	Drosophila ditch	sweeping Paisonia Leaves	DOFAW I1012; Koke'e state park K2017-2015; I1012; NARS special use; Kaua'i island forest reserves KPI-2017-114	4/16/2017	N22° 04.795' W159° 40.448'
002C	*Drosophila nanella*	Koke'e State Park	Drosophila ditch	sweeping Paisonia Leaves	DOFAW I1012; Koke'e state park K2017-2015; I1012; NARS special use; Kaua'i island forest reserves KPI-2017-114	4/13/2017	N22° 04.795' W159° 40.448'
032A	*Drosophila nanella*	Koke'e State Park	Drosophila ditch	sweeping Paisonia Leaves	DOFAW I1012; Koke'e state park K2017-2015; I1012; NARS special use; Kaua'i island forest reserves KPI-2017-114	4/16/2017	N22° 04.795' W159° 40.448'
002D	*Drosophila nanella*	Koke'e State Park	Drosophila ditch	sweeping Paisonia Leaves	DOFAW I1012; Koke'e state park K2017-2015; I1012; NARS special use; Kaua'i island forest reserves KPI-2017-114	4/13/2017	N22° 04.795' W159° 40.448'
024B	*Drosophila primaeva*	Koke'e State Park	Drosophila gulch	baits	DOFAW I1012; Koke'e state park K2017-2015; I1012; NARS special use; Kaua'i island forest reserves KPI-2017-114	4/16/2017	N22° 08.914' W159° 38.175'
021A	*Drosophila atroscutellata*	Koke'e State Park	Nualolo trail	sweeping Corynocarpus sp leaves	DOFAW I1012; Koke'e state park K2017-2015; I1012; NARS special use; Kaua'i island forest reserves KPI-2017-114	4/15/2017	N22° 07.801' W159° 39.617'
029D	*Drosophila atroscutellata*	Koke'e State Park	Nualolo trail	sweeping Corynocarpus sp leaves	DOFAW I1012; Koke'e state park K2017-2015; I1012; NARS special use; Kaua'i island forest reserves KPI-2017-114	4/16/2017	N22° 07.801' W159° 39.617'
029A	*Drosophila atroscutellata*	Koke'e State Park	Nualolo trail	sweeping Corynocarpus sp leaves	DOFAW I1012; Koke'e state park K2017-2015; I1012; NARS special use; Kaua'i island forest reserves KPI-2017-114	4/16/2017	N22° 07.801' W159° 39.617'
016B	*Drosophila picticornis*	Koke'e State Park	Nualolo trail	baits	DOFAW I1012; Koke'e state park K2017-2015; I1012; NARS special use; Kaua'i island forest reserves KPI-2017-114	4/15/2017	N22° 07.801' W159° 39.617'
016C	*Drosophila picticornis*	Koke'e State Park	Nualolo trail	baits	DOFAW I1012; Koke'e state park K2017-2015; I1012; NARS special use; Kaua'i island forest reserves KPI-2017-114	4/15/2017	N22° 07.801' W159° 39.617'
028A	*Drosophila picticornis*	Koke'e State Park	Nualolo trail	baits	DOFAW I1012; Koke'e state park K2017-2015; I1012; NARS special use; Kaua'i island forest reserves KPI-2017-114	4/15/2017	N22° 07.801' W159° 39.617'
020B	*Scaptomyza varipicta*	Koke'e State Park	Nualolo trail	sweeping Cheirodendron sp leaves	DOFAW I1012; Koke'e state park K2017-2015; I1012; NARS special use; Kaua'i island forest reserves KPI-2017-114	4/15/2017	N22° 08.336' W159° 40.479'

Table A: Field collection information for sequenced specimens. *(continued)*

individual	species	general site	locality	collection method	permit	collection date	GPS
020C	*Scaptomyza varipicta*	Koke'e State Park	Nualolo trail	sweeping Cheiroden-dron sp leaves	DOFAW I1012; Koke'e state park K2017-2015; I1012; NARS special use; Kaua'i island forest reserves KPI-2017-114	4/15/2017	N22° 08.336' W159° 40.479'
020D	*Scaptomyza varipicta*	Koke'e State Park	Nualolo trail	sweeping Cheiroden-dron sp leaves	DOFAW I1012; Koke'e state park K2017-2015; I1012; NARS special use; Kaua'i island forest reserves KPI-2017-114	4/15/2017	N22° 08.336' W159° 40.479'
020A	*Scaptomyza varipicta*	Koke'e State Park	Nualolo trail	sweeping Cheiroden-dron sp leaves	DOFAW I1012; Koke'e state park K2017-2015; I1012; NARS special use; Kaua'i island forest reserves KPI-2017-114	4/15/2017	N22° 08.336' W159° 40.479'
8.0-1	*Drosophila macrothrix*	Hawai'i Volcanoes National Park	Ola'a tract, pole 44	baits	DOFAW I1012; HAVO-2017-SCI-0017	5/9/2016	N19° 27.722' W155° 14.875'
8.0-2	*Drosophila macrothrix*	Hawai'i Volcanoes National Park	Ola'a tract, pole 44	baits	DOFAW I1012; HAVO-2017-SCI-0017	5/9/2016	N19° 27.722' W155° 14.875'
8.0-3	*Drosophila macrothrix*	Hawai'i Volcanoes National Park	Ola'a tract, pole 44	baits	DOFAW I1012; HAVO-2017-SCI-0017	5/9/2016	N19° 27.722' W155° 14.875'
055A	*Drosophila macrothrix*	Hawai'i Volcanoes National Park	Ola'a tract, pole 44	baits	DOFAW I1012; HAVO-2017-SCI-0017	4/17/2017	N19° 27.722' W155° 14.875'
7.1-1	*Drosophila sproati*	Hawai'i Volcanoes National Park	Ola'a tract, pole 44	baits	DOFAW I1012; HAVO-2017-SCI-0017	5/9/2016	N19° 27.722' W155° 14.875'
7.2-1	*Drosophila sproati*	Hawai'i Volcanoes National Park	Ola'a tract, pole 44	baits	DOFAW I1012; HAVO-2017-SCI-0017	5/9/2016	N19° 27.722' W155° 14.875'
106B	*Drosophila sproati*	Hawai'i Volcanoes National Park	Ola'a tract, pole 44	baits	DOFAW I1012; HAVO-2017-SCI-0017	5/29/2017	N19° 27.722' W155° 14.875'
106A	*Drosophila sproati*	Hawai'i Volcanoes National Park	Ola'a tract, pole 44	baits	DOFAW I1012; HAVO-2017-SCI-0017	5/29/2017	N19° 27.722' W155° 14.875'
043C	*Drosophila tanythrix*	Hawai'i Volcanoes National Park	Ola'a tract, pole 44	baits	DOFAW I1012; HAVO-2017-SCI-0017	4/18/2017	N19° 27.722' W155° 14.875'
056A	*Drosophila tanythrix*	Hawai'i Volcanoes National Park	Ola'a tract, pole 44	baits	DOFAW I1012; HAVO-2017-SCI-0017	4/17/2017	N19° 27.722' W155° 14.875'
40.2-1	*Drosophila tanythrix*	Hawai'i Volcanoes National Park	Ola'a tract, pole 44	baits	DOFAW I1012; HAVO-2017-SCI-0017	5/9/2016	N19° 27.722' W155° 14.875'
043D	*Drosophila tanythrix*	Hawai'i Volcanoes National Park	Ola'a tract, pole 44	baits	DOFAW I1012; HAVO-2017-SCI-0017	4/18/2017	N19° 27.722' W155° 14.875'
008A	*Drosophila primaeva*	Koke'e State Park	Pihea trail	baits	DOFAW I1012; Koke'e state park K2017-2015; I1012; NARS special use; Kaua'i island forest reserves KPI-2017-114	4/14/2017	N22° 08.799' W159° 37.074'
012A	*Drosophila primaeva*	Koke'e State Park	Pihea trail	baits	DOFAW I1012; Koke'e state park K2017-2015; I1012; NARS special use; Kaua'i island forest reserves KPI-2017-114	4/14/2017	N22° 08.799' W159° 37.074'

Table A: Field collection information for sequenced specimens. *(continued)*

individual	species	general site	locality	collection method	permit	collection date	GPS
008D	*Drosophila primaeva*	Koke'e State Park	Pihea trail	baits	DOFAW I1012; Koke'e state park K2017-2015; I1012; NARS special use; Kaua'i island forest reserves KPI-2017-114	4/14/2017	N22° 08.799' W159° 37.074'
CFC	*Scaptomya varia*	Koke'e State Park	Pihea trail	collected rotting Clermontia sp flowers	DOFAW I1012; Koke'e state park K2017-2015; I1012; NARS special use; Kaua'i island forest reserves KPI-2017-114	4/14/2017	N22° 08.799' W159° 37.074'
CFA	*Scaptomya varia*	Koke'e State Park	Pihea trail	collected rotting Clermontia sp flowers	DOFAW I1012; Koke'e state park K2017-2015; I1012; NARS special use; Kaua'i island forest reserves KPI-2017-114	4/14/2017	N22° 08.799' W159° 37.074'
CFB	*Scaptomya varia*	Koke'e State Park	Pihea trail	collected rotting Clermontia sp flowers	DOFAW I1012; Koke'e state park K2017-2015; I1012; NARS special use; Kaua'i island forest reserves KPI-2017-114	4/14/2017	N22° 08.799' W159° 37.074'

Table B: DNA barcoding for identification of females.

individual	sample	species match	reference male	external reference sequence	barcode sequence used for final identification	notes
040B	040Bb	*D. mimica*	yes	yes	16S	
040C	040Ctxt	*D. mimica*	yes	yes	16S	
040D	040Db	*D. mimica*	yes	yes	16S	
18.0-3	18.0.17	*D. mimica*	yes	yes	16S	
CFA	CFAb	*S. varia*	yes	yes	COI	
CFB	CFBb	*S. varia*	yes	yes	COI	
CFC	CFCb	*S. varia*	yes	yes	COI	
088A	088Ab	*S. cyrtandrae*	none	yes	COII	
088B	088Bb	*S. cyrtandrae*	none	yes	COII	
088C	088Cb	*S. cyrtandrae*	none	yes	COII	
021A	021Ab	*D. atroscutellata*	yes	yes	COII	
029A	029Atxt	*D. atroscutellata*	yes	yes	COII	
029D	029Db	*D. atroscutellata*	yes	yes	COII	
002C	002Cb	*D. nanella*	yes	yes	16S, COII	
002D	002Dtxt	*D. nanella*	yes	yes	16S, COII	
032A	032Ab	*D. nanella*	yes	yes	16S, COII	
032B	032Bb	*D. nanella*	yes	yes	16S, COII	
106A	106Atxt	*D. sproati*	none	none	COII	matched to other females, morphology is distinctive for females in this species
106B	106Bb	*D. sproati*	none	none	COII	matched to other females, morphology is distinctive for females in this species
7.1-1	7.1.4	*D. sproati*	none	none	COII	matched to other females, morphology is distinctive for females in this species
043C	043Cb	*D. tanythrix*	yes	yes	COII	barcode sequences for D. cognata and D. yooni males suggest hidden complexity in this group
043D	043Dtxt	*D. tanythrix*	yes	yes	COII	barcode sequences for D. cognata and D. yooni males suggest hidden complexity in this group
056A	056Ab	*D. tanythrix*	yes	yes	COII	barcode sequences for D. cognata and D. yooni males suggest hidden complexity in this group
40.2-1	40.2.2	*D. tanythrix*	yes	yes	COII	barcode sequences for D. cognata and D. yooni males suggest hidden complexity in this group
020A	020Atxt	*S. varipicta*	yes	yes	COII	
020B	020Bb	*S. varipicta*	yes	yes	COII	
020C	020Cb	*S. varipicta*	yes	yes	COII	
020D	020Db	*S. varipicta*	yes	yes	COII	

Table B: DNA barcoding for identification of females. *(continued)*

individual	sample	species match	reference male	external reference sequence	barcode sequence used for final identification	notes
16.1-1	16.1.4	*D. cf dives*	none	none	16S, COII	found no matching reference sequence and no males were caught
16.2-1	16.2.4	*D. cf dives*	none	none	16S, COII	found no matching reference sequence and no males were caught

Table C: Paired-end sequencing read counts.

species	individual ID	sample ID	tissue	reads - round 1	reads - round 2	reads - round 3	total reads
D. atroscutellata	029A	029Atxt	whole fly	12,520,922	7,801,177	19,925,042	40,247,141
D. cf dives	16.1-1	16.1.1	ovary	16,808,131			16,808,131
D. cf dives	16.1-1	16.1.2	head	18,215,227			18,215,227
D. cf dives	16.1-1	16.1.4	carcass		8,773,601		8,773,601
D. macrothrix	055A	055Atxt	whole fly	10,336,762	10,313,394	19,643,740	40,293,896
D. macrothrix	8.0-2	8.0.6	ovary	8,886,609			8,886,609
D. mimica	040C	040Ctxt	whole fly	9,471,290	8,887,955	15,751,511	34,110,756
D. nanella	002D	002Dtxt	whole fly	10,833,205	7,350,705	17,211,801	35,395,711
D. picticornis	025A	025Atxt	whole fly	10,085,602	10,177,523	16,172,455	36,435,580
D. primaeva	008D	008Dtxt	whole fly	9,129,075	7,583,577	13,937,132	30,649,784
D. sproati	106A	106Atxt	whole fly	7,432,370	6,026,569	10,237,744	23,696,683
D. tanythrix	043D	043Dtxt	whole fly	11,293,054	8,833,878	15,667,639	35,794,571
S. cyrtandrae	088B	088Bb	carcass		9,166,453		9,166,453
S. cyrtandrae	088B	088Bn	head		8,796,864		8,796,864
S. cyrtandrae	088B	088Bo	ovary		9,763,204		9,763,204
S. cyrtandrae	088C	088Cb	carcass	11,218,315			11,218,315
S. cyrtandrae	088C	088Cn	head	7,670,354			7,670,354
S. cyrtandrae	088C	088Co	ovary	7,145,307			7,145,307
S. varia	CFB	CFBb	carcass		6,672,052		6,672,052
S. varia	CFB	CFBn	head		6,311,203		6,311,203
S. varia	CFB	CFBo	ovary		12,672,693		12,672,693
S. varia	CFC	CFCb	carcass	6,941,585			6,941,585
S. varia	CFC	CFCn	head	8,853,906			8,853,906
S. varia	CFC	CFCo	ovary	8,161,304			8,161,304
S. varipicta	020A	020Atxt	whole fly	7,690,349	8,004,757	14,380,332	30,075,438

Table D: Single-end sequencing read counts.

species	individual ID	sample ID	tissue	notes	reads - round 1	reads - round 2	reads - round 3	reads - round 4	total reads
D. atroscutellata	021A	021Ab	carcass	ovipositor removed			14,147,182		14,147,182
D. atroscutellata	021A	021An	head				14,091,512		14,091,512
D. atroscutellata	021A	021Ao	ovary				17,225,774		17,225,774
D. atroscutellata	029D	029Db	carcass	ovipositor removed				13,664,824	13,664,824
D. atroscutellata	029D	029Dn	head					18,613,630	18,613,630
D. atroscutellata	029D	029Do	ovary	no vitel-logenic eggs				19,916,293	19,916,293
D. cf dives	16.1-1	16.1.1	ovary		12,041,362				12,041,362
D. cf dives	16.1-1	16.1.2	head		18,382,021				18,382,021
D. cf dives	16.1-1	16.1.4	carcass		21,990,829				21,990,829
D. cf dives	16.2-1	16.2.1	ovary		16,508,284				16,508,284
D. cf dives	16.2-1	16.2.2	head			11,288,812			11,288,812
D. cf dives	16.2-1	16.2.4	carcass		14,384,696				14,384,696
D. macrothrix	8.0-1	8.0.1	ovary		625,317	337,147			962,464
D. macrothrix	8.0-1	8.0.2	head		28,027,155	12,695,316			40,722,471
D. macrothrix	8.0-1	8.0.4	carcass		18,125,412				18,125,412
D. macrothrix	8.0-2	8.0.6	ovary		15,583,003				15,583,003
D. macrothrix	8.0-2	8.0.7	head					12,639,355	12,639,355
D. macrothrix	8.0-2	8.0.9	carcass		42,026,251				42,026,251
D. macrothrix	8.0-3	8.0.12	carcass		29,231,317				29,231,317
D. macrothrix	8.0-3	8.0.15	ovary		35,169,909				35,169,909
D. macrothrix	8.0-3	8.0.16	head		17,682,909				17,682,909
D. mimica	040B	040Bb	carcass					13,288,927	13,288,927

Table D: Single-end sequencing read counts. *(continued)*

species	individual ID	sample ID	tissue	notes	reads - round 1	reads - round 2	reads - round 3	reads - round 4	total reads
D. mimica	040B	040Bn	head					12,473,218	12,473,218
D. mimica	040B	040Bo	ovary					14,622,979	14,622,979
D. mimica	040D	040Db	carcass				20,653,357		20,653,357
D. mimica	040D	040Db	head				12,923,155		12,923,155
D. mimica	040D	040Db	ovary				17,181,968		17,181,968
D. mimica	18.0-3	18.0.14	ovary				13,450,864	17,450,051	30,900,915
D. mimica	18.0-3	18.0.15	head	sequencing failed					0
D. mimica	18.0-3	18.0.17	carcass		19,971,465			11,948,038	31,919,503
D. nanella	002C	002Cb	carcass			15,668,215			15,668,215
D. nanella	002C	002Cn	head			18,850,083			18,850,083
D. nanella	002C	002Co	ovary			19,143,364			19,143,364
D. nanella	032A	032Ab	carcass	ovipositor removed			16,985,268		16,985,268
D. nanella	032A	032An	head				15,262,003		15,262,003
D. nanella	032A	032Ao	ovary				26,566,152		26,566,152
D. nanella	032B	032Bb	carcass	ovipositor removed				13,166,018	13,166,018
D. nanella	032B	032Bn	head					7,720,333	7,720,333
D. nanella	032B	032Bo	ovary					10,507,831	10,507,831
D. picticornis	016B	016Bb	carcass					13,944,238	13,944,238
D. picticornis	016B	016Bn	head					12,710,348	12,710,348
D. picticornis	016B	016Bo	ovary					8,261,788	8,261,788
D. picticornis	016C	016Cb	carcass	ovipositor removed		19,183,647			19,183,647
D. picticornis	016C	016Cn	head			20,597,014			20,597,014
D. picticornis	016C	016Co	ovary			18,242,638			18,242,638
D. picticornis	028A	028Ab	carcass	ovipositor removed			17,704,561	21,917,683	39,622,244
D. picticornis	028A	028An	head				15,060,854	12,034,753	27,095,607
D. picticornis	028A	028Ao	ovary				25,988,121	10,261,716	36,249,837
D. primaeva	008A	008Ab	carcass	ovipositor removed				21,917,683	21,917,683
D. primaeva	008A	008An	head					12,034,753	12,034,753
D. primaeva	008A	008Ao	ovary					10,261,716	10,261,716
D. primaeva	012A	012Ab	carcass			16,297,595			16,297,595
D. primaeva	012A	012An	head			12,515,877			12,515,877
D. primaeva	012A	012Ao	ovary	all oocytes were at the same stage		14,945,335			14,945,335
D. primaeva	024B	024Bb	carcass				21,578,797		21,578,797
D. primaeva	024B	024Bn	head				13,850,246		13,850,246
D. primaeva	024B	024Bo	ovary	all oocytes were at the same stage			12,001,961		12,001,961
D. sproati	106B	106Bb	carcass			17,653,753			17,653,753
D. sproati	106B	106Bn	ovary			10,220,778			10,220,778
D. sproati	106B	106Bo	head			15,615,762			15,615,762
D. sproati	7.1-1	7.1.1	ovary		9,527,229				9,527,229
D. sproati	7.1-1	7.1.2	head	sequencing failed					0
D. sproati	7.1-1	7.1.4	carcass		13,815,824				13,815,824
D. sproati	7.2-1	7.2.1	carcass		22,462,042				22,462,042
D. sproati	7.2-1	7.2.2	head		21,654,908				21,654,908
D. sproati	7.2-1	7.2.4	ovary		31,064,408				31,064,408
D. tanythrix	043C	043Cb	carcass			11,381,085			11,381,085
D. tanythrix	043C	043Cn	head			9,395,340			9,395,340
D. tanythrix	043C	043Co	ovary			13,769,669			13,769,669
D. tanythrix	056A	056Ab	carcass				20,904,414		20,904,414
D. tanythrix	056A	056An	head				13,824,612		13,824,612
D. tanythrix	056A	056Ao	ovary				17,772,798		17,772,798
D. tanythrix	40.2-1	40.2.1	ovary		30,223,622				30,223,622
D. tanythrix	40.2-1	40.2.2	head		27,274,868		17,405,688	17,790,177	62,470,733
D. tanythrix	40.2-1	40.2.4	carcass		22,021,646	16,772,624			38,794,270
S. cyrtandrae	088A	088Ab	carcass			19,038,552			19,038,552
S. cyrtandrae	088A	088An	head			12,179,789			12,179,789
S. cyrtandrae	088A	088Ao	ovary			13,439,568			13,439,568
S. cyrtandrae	088B	088Bb	carcass				11,543,268		11,543,268
S. cyrtandrae	088B	088Bn	head				12,904,077		12,904,077
S. cyrtandrae	088B	088Bo	ovary				11,569,554		11,569,554
S. cyrtandrae	088C	088Cb	carcass					15,301,343	15,301,343
S. cyrtandrae	088C	088Cn	head					8,191,593	8,191,593

Table D: Single-end sequencing read counts. *(continued)*

species	individual ID	sample ID	tissue	notes	reads - round 1	reads - round 2	reads - round 3	reads - round 4	total reads
S. cyrtandrae	088C	088Co	ovary					7,158,247	7,158,247
S. varia	CFA	CFAb	carcass			14,094,153			14,094,153
S. varia	CFA	CFAn	head			10,655,605			10,655,605
S. varia	CFA	CFAo	ovary			10,702,817			10,702,817
S. varia	CFB	CFBb	carcass				8,049,520		8,049,520
S. varia	CFB	CFBn	head				7,357,905		7,357,905
S. varia	CFB	CFBo	ovary				12,557,066		12,557,066
S. varia	CFC	CFCb	carcass					11,159,366	11,159,366
S. varia	CFC	CFCn	head					11,517,456	11,517,456
S. varia	CFC	CFCo	ovary					10,874,670	10,874,670
S. varipicta	020B	020Bb	carcass			8,132,403			8,132,403
S. varipicta	020B	020Bn	head			14,331,603			14,331,603
S. varipicta	020B	020Bo	ovary			8,192,636			8,192,636
S. varipicta	020C	020Cb	carcass	ovipositor removed			16,584,774		16,584,774
S. varipicta	020C	020Cn	head				13,783,546		13,783,546
S. varipicta	020C	020Co	ovary				17,539,765		17,539,765
S. varipicta	020D	020Db	carcass	ovipositor removed				19,358,586	19,358,586
S. varipicta	020D	020Dn	head					19,832,422	19,832,422
S. varipicta	020D	020Do	ovary					13,226,137	13,226,137

Table E: Number of transcripts per homology group per species.

species	homology groups	groups with more than one transcript	max num. transcripts per group	ave num. transcripts per group
D. atroscutellata	3,939	1,211	8	1.40
D. cf dives	4,782	966	8	1.28
D. macrothrix	4,061	1,164	8	1.39
D. mimica	4,278	1,344	8	1.42
D. nanella	4,790	1,271	7	1.36
D. picticornis	2,769	784	6	1.37
D. primaeva	3,274	935	7	1.39
D. sproati	3,380	767	8	1.30
D. tanythrix	3,618	1,015	7	1.38
S. cyrtandrae	4,966	1,283	7	1.35
S. varia	4,733	1,109	12	1.32
S. varipicta	4,696	1,195	8	1.35

2.2 Core genes

Table F: Core ovary genes.

Dmel parent gene ID	Dmel parent gene symbol	transcripts in homology group	homology group Dmel sequence symbols
FBgn0250816	AGO3	none	none
FBgn0087040	alphaTub67C	none	none
FBgn0266111	ana3	none	none
FBgn0028343	Ankle2	some	Ankle2
FBgn0041164	armi	none	none
FBgn0029094	asf1	all	asf1
FBgn0005386	ash1	none	none
FBgn0000140	asp	none	none
FBgn0000182	BicC	none	none
FBgn0033155	Br140	some	Br140
FBgn0010300	brat	some	brat
FBgn0263855	BubR1	some	BubR1
FBgn0039680	Cap	none	none
FBgn0027512	CG10254	none	none
FBgn0037021	CG11399	some	CG11399
FBgn0038968	CG12499	none	none
FBgn0031070	CG12702	none	none
FBgn0039640	CG14516	none	none
FBgn0034498	CG16868	none	none
FBgn0052344	CG32344	none	none
FBgn0031875	CG3430	some	CG3430
FBgn0250754	CG42232	none	none
FBgn0029733	CG6927	some	CG6927;pHCl;SecCl

Table F: Core ovary genes. *(continued)*

Dmel parent gene ID	Dmel parent gene symbol	transcripts in homology group	homology group Dmel sequence symbols
FBgn0034187	CG6967	some	CG6701;CG6967
FBgn0031947	CG7154	some	CG7154
FBgn0034073	CG8414	all	CG8414
FBgn0037664	CG8420	none	none
FBgn0031769	CG9135	some	CG9135
FBgn0000307	chif	none	none
FBgn0044324	Chro	none	none
FBgn0261016	clos	some	no Dmel match
FBgn0033890	Ctf4	some	Ctf4
FBgn0000392	cup	none	none
FBgn0000404	CycA	some	CycA
FBgn0000405	CycB	some	CycB
FBgn0015625	CycB3	some	CycB3
FBgn0010382	CycE	some	CycE
FBgn0039016	Dcr	some	no Dmel match
FBgn0000463	Dl	none	none
FBgn0262619	DNAlig1	none	none
FBgn0259113	DNApol	none	none
FBgn0264326	DNApol	none	none
FBgn0002905	DNApol	none	none
FBgn0263600	DNApol	none	none
FBgn0002183	dre4	none	none
FBgn0000996	dup	some	dup;Pms2
FBgn0087008	e(y)3	some	e(y)3
FBgn0087008	e(y)3	some	no Dmel match
FBgn0000615	exu	some	exu
FBgn0033354	FANCI	some	CG33680;FANCI
FBgn0005390	fs(1)M3	none	none
FBgn0004650	fs(1)N	none	none
FBgn0001085	fz	all	fz
FBgn0283682	Ge	none	none
FBgn0015391	glu	none	none
FBgn0266580	Gp210	none	none
FBgn0261278	grp	some	grp
FBgn0283499	InR	none	none
FBgn0011604	Iswi	none	none
FBgn0015396	jumu	some	jumu
FBgn0030268	Klp10A	some	Klp10A;Klp59C;Klp59D
FBgn0038476	kuk	none	none
FBgn0034657	LBR	some	LBR
FBgn0019686	lok	all	lok
FBgn0283521	lola	some	no Dmel match
FBgn0283521	lola	some	pre
FBgn0039972	Marf1	some	Marf1
FBgn0025743	mbt	some	mbt
FBgn0014861	Mcm2	some	Mcm2
FBgn0284442	Mcm3	some	Mcm3
FBgn0017577	Mcm5	none	none
FBgn0025815	Mcm6	none	none
FBgn0020633	Mcm7	none	none
FBgn0004419	me31B	all	me31B
FBgn0261786	mi	none	none
FBgn0036486	Msh6	some	Msh6
FBgn0010431	mtrm	some	no Dmel match
FBgn0002872	mu2	some	mu2
FBgn0002873	mud	none	none
FBgn0002878	mus101	none	none
FBgn0002924	ncd	none	none
FBgn0029970	Nek2	some	Nek2
FBgn0032848	nesd	all	nesd
FBgn0002962	nos	all	nos
FBgn0039559	NSD	none	none
FBgn0021761	Nup154	none	none
FBgn0033766	Nup188	some	Nup188
FBgn0031078	Nup205	none	none
FBgn0034310	Nup75	all	Nup75
FBgn0004882	orb	some	orb
FBgn0003015	osk	none	none
FBgn0003028	ovo	some	no Dmel match
FBgn0003044	Pcl	none	none
FBgn0261811	pico	some	pico
FBgn0004872	piwi	none	none
FBgn0036354	Poc1	some	Poc1
FBgn0003124	polo	some	polo
FBgn0003165	pum	some	tna
FBgn0261987	Pxt	none	none
FBgn0003187	qua	none	none

Table F: Core ovary genes. *(continued)*

Dmel parent gene ID	Dmel parent gene symbol	transcripts in homology group	homology group Dmel sequence symbols
FBgn0003346	RanGAP	all	RanGAP
FBgn0039644	rdog	none	none
FBgn0264493	rdx	some	rdx
FBgn0040290	RecQ4	some	CG6888;Jafrac1;Jafrac2;Prx3;RecQ4
FBgn0020379	Rfx	some	Rfx
FBgn0034249	RhoGAP54D	some	RhoGAP54D
FBgn0034249	RhoGAP54D	some	dgt3
FBgn0050085	Rif1	some	Rif1
FBgn0250850	rig	none	none
FBgn0011703	RnrL	some	RnrL
FBgn0003268	rod	some	rod
FBgn0025802	Sbf	none	none
FBgn0032475	Sfmbt	none	none
FBgn0003401	shu	some	shu
FBgn0051163	SKIP	some	SKIP
FBgn0016070	smg	some	CG5280;smg
FBgn0016070	smg	some	no Dmel match
FBgn0037025	Spc105R	none	none
FBgn0027500	spd	none	none
FBgn0003483	spn	none	none
FBgn0033348	Spt	some	Spt;Sry
FBgn0262733	Src64B	none	none
FBgn0002466	sti	none	none
FBgn0052676	stx	some	stx
FBgn0003655	swa	all	swa
FBgn0031030	Tao	none	none
FBgn0003701	thr	none	none
FBgn0284220	Top2	none	none
FBgn0033636	tou	none	none
FBgn0041775	tral	some	tral
FBgn0086356	tum	some	Echs1;RacGAP84C;tum
FBgn0260780	wisp	some	wisp
FBgn0039338	XNP	some	XNP
FBgn0039338	XNP	some	no Dmel match
FBgn0004649	yl	none	none
FBgn0259789	zld	some	no Dmel match

Table G: Core head genes.

Dmel parent gene ID	Dmel parent gene symbol	transcripts in homology group	homology group Dmel sequence symbols
FBgn0261788	Ank2	none	none
FBgn0000120	Arr1	all	no Dmel match
FBgn0000121	Arr2	some	no Dmel match
FBgn0000206	boss	all	no Dmel match
FBgn0264386	Ca	none	none
FBgn0263111	cac	none	none
FBgn0015609	CadN	none	none
FBgn0053653	Cadps	none	none
FBgn0013759	CASK	none	none
FBgn0035720	CG10077	none	none
FBgn0039927	CG11155	none	none
FBgn0033250	CG14762	some	no Dmel match
FBgn0052432	CG32432	none	none
FBgn0263077	CG43340	none	none
FBgn0029834	CG5937	none	none
FBgn0035253	CG7971	some	no Dmel match
FBgn0040502	CG8343	some	no Dmel match
FBgn0267435	chp	none	none
FBgn0000346	comt	none	none
FBgn0285925	Fas1	some	no Dmel match
FBgn0259108	futsch	none	none
FBgn0027575	GABA	some	no Dmel match
FBgn0004435	Galphaq	some	no Dmel match
FBgn0004623	Gbeta76C	some	no Dmel match
FBgn0010114	hig	some	no Dmel match
FBgn0031294	IA	some	no Dmel match
FBgn0004784	inaC	none	none
FBgn0261794	kcc	some	no Dmel match
FBgn0000464	Lar	none	none
FBgn0267429	lovit	some	no Dmel match
FBgn0085434	NaCP60E	none	none
FBgn0002938	ninaC	none	none
FBgn0002940	ninaE	some	no Dmel match
FBgn0262738	norpA	none	none

Table G: Core head genes. *(continued)*

Dmel parent gene ID	Dmel parent gene symbol	transcripts in homology group	homology group Dmel sequence symbols
FBgn0032946	nrv3	some	no Dmel match
FBgn0038975	Nrx	some	no Dmel match
FBgn0259994	OtopLa	some	no Dmel match
FBgn0285944	para	none	none
FBgn0011693	Pdh	some	no Dmel match
FBgn0264598	PsGEF	some	no Dmel match
FBgn0263102	psq	some	no Dmel match
FBgn0003249	Rh3	some	no Dmel match
FBgn0003250	Rh4	all	no Dmel match
FBgn0004574	Rop	none	none
FBgn0003392	shi	none	none
FBgn0267001	Ten	none	none
FBgn0030412	Tomosyn	some	no Dmel match
FBgn0003861	trp	none	none
FBgn0025726	unc	none	none
FBgn0267002	unc	none	none
FBgn0038693	unc79	none	none
FBgn0039536	unc80	none	none

References

1. Leader DP, Krause SA, Pandit A, Davies SA, Dow JAT. FlyAtlas 2: A new version of the *Drosophila melanogaster* expression atlas with RNA-seq, miRNA-seq and sex-specific data. Nucleic Acids Research. 2018;46: D809–D815.

2. Larkin A, Marygold SJ, Antonazzo G, Attrill H, Dos Santos G, Garapati PV, et al. FlyBase: Updates to the *Drosophila* melanogaster knowledge base. Nucleic Acids Research. 2021;49: D899–D907.
