# Supplementary material for: The evolution of ovary-biased gene expression in Hawaiian Drosophila
Source: PLoS Genet. 2023 Jan 23;19(1):e1010607. doi: 10.1371/journal.pgen.1010607 (PMC9894553; doi:10.1371/journal.pgen.1010607)
Supplement: Attachment — Submitted filename: 221106_ReviewerResponse_HawaiianDrosophilaExpression.pdf [file pgen.1010607.s002.pdf]

November 6, 2022

Dear Dr. Kopp and Dr. Bomblies,

We thank the reviewers for their constructive reviews of our originally submitted manuscript. We have addressed all of their concerns, as detailed in our point-by point response below. We hope that you and the reviewers will find the revised manuscript suitable for publication.

Our responses to all verbatim points raised by reviewers are provided below in blue text. Changes to the manuscript made in response to these reviews are indicated in red text.

We look forward to hearing from you.

Sincerely,

Samuel H. Church

The manuscript was fully evaluated at the editorial level and by independent peer reviewers. The reviewers appreciated the attention to an important problem, but raised some substantial concerns about the current manuscript. Reviewer #2, in particular, raises some questions about the robustness of statistical inferences based on pairwise correlations, and suggests potential alternative approaches that you may wish to consider. Confirming the predictive value of the co-evolutionary analysis (e.g., in the inference of "new networks of genes that share evolutionary trajectories") by validation experiments, as that reviewer suggests, would of course be great, but I think that goes beyond the scope of this paper. Demonstrating the robustness of the co-evolutionary analysis itself seems more important to this study, especially given the small magnitude of some of the differences you observe. I suggest paying particular attention to the major point 1 and minor points 1 and 4 of the 2nd reviewer. As for the "why reconstruct bias" question raised by Reviewer #3, I believe that has been discussed in Casey's previous papers, but a more thorough recap of that logic would help keep the reviewers and readers on board.

RESPONSE: We thank the editors for a very helpful review process. We have addressed the reviewers' concerns, as detailed below. In particular, we have incorporated a new, stricter, analysis of correlations (l. 291-295, 613-615), and we have updated the text to clarify our averaging procedure (l. 212-213), and our choice of expression bias rather than absolute expression magnitude (l. 124-126, 571-573).

Reviewer #1: Church et al. describe changes in gene expression across a panel of the Drosophilidae. This was enjoyable to read and a well thought through manuscript. An interesting finding was that species variation overwhelms tissue as you move out in evolutionary time. I think this is an important contrast in the literature to many of the mammalian organ studies.

RESPONSE: We thank the reviewer for their helpful review and interest in our work.

Major comments:

Some comments in the discussion about why this does look different than many of the mammalian studies is warranted. Generation time, population size, ect. What else do the authors think? Would you expect this finding in all species if you go out far enough in evolutionary time?

RESPONSE: We thank the reviewer for this suggestion and have added the following summary statement to the discussion (l. 335-336):

"In summary, consistent with recent findings in mammals (Breschi et al, 2016), our study shows that our expectation about the interspecific similarity of tissues should decrease as the phylogenetic distance increases, and the rate of that decrease will depend on the tissue in question."

In answer to the question, we expect to observe this same finding for any set of species, if you go far enough in evolutionary time. We consider these findings are largely consistent with what has been shown in mammals, specifically that the similarity of tissues depends on the evolutionary distance (often relatively less distant between mammals that other study organisms) and the tissues considered.

Minor comments:

1. The font on many of the figures is really small (Figure 2 is an example).

RESPONSE: We increased the font size in all figures, including the text labels in Figure 2.

2. The methods from ref 44 should be summarized in the Methods section (pg 17).

RESPONSE: We added a summary of those phylogenetic methods, as suggested (section 5.6).

3. Estimated times of divergence would be nice in Figure 1A – gave some perspective on how this compares with other, similar, analyses.

RESPONSE: We added estimated divergence times, as suggested.

Reviewer #2: Overview:

In this work, the authors present an analysis of tissue-biased gene expression in a phylogenetic context for head and ovary relative to carcass in female Hawaiian Drosophila from twelve species. Four primary analyses are presented, with the results from the correlation analysis made available as a resource via github as in an interactive and queryable format. First, expression is compared across species to identify genes with conserved ovary-biased (or head-biased) expression. Second, an analysis comparing sources of variation, tissue and species, is conducted in order to identify the phylogenetic distance at which species differences within tissue exceed tissue differences within species. Third, gains/losses of tissue bias are examined based on scaled changes in tissue bias (relative to predicted ancestral bias) along the phylogeny. Finally, the Pearson correlation of scaled changes in tissue bias between genes is examined with the goal of identifying co-evolutionary changes in gene expression that may indicate functional relationships between genes. These pairwise correlations are made available to support an approach in which genes showing shared patterns of expression changes may be classified as putatively interacting – a proposed resource for future hypothesis testing.

The main strength of this manuscript is the novel data set and focus on evolution of tissue-biased expression. Collection of this dataset represents a large amount of effort and resources and is of interest to the evo devo community. The manuscript itself is centered around the dataset and presenting an exploration of several ways that evolution of tissue-biased gene expression can be examined in the context of phylogenetic history. Another theme is a more quantitative approach to caveats related to the use of model organism data to make inferences across large evolutionary distances. The focus on tissue bias in a phylogenetic context has the potential to yield novel findings and be of interest to a broad audience.

There is no doubt that these data are a valuable contribution to understanding evolution in the Hawaiian Drosophila and expression evolution at the tissue level more broadly, however there are significant weaknesses in the current approach and manuscript. The largest issue is a lack of the ability to assess confidence in the information content and practical application of the correlation analysis. The small average difference between groups based on known/unknown functional relationships and the large degree of variation are acknowledged by the authors, but there is not an attempt to further assess if/how this can be a useful tool. Some options would be to try alternative analytical approaches, to examine Pearson correlation in a network context more quantitatively, and/or to use validation to assess the usefulness of this approach in identifying candidates. More grounding in the literature via citation and discussion would also aid in highlighting the relevance of the findings and clarify their context in relation to current scientific knowledge. In addition, findings that are relevant to the unique biology and evolutionary history of the Hawaiian Drosophilidae and of the ovary are not a major theme and would be of broad interest.

RESPONSE: We thank the reviewer for their close reading of our manuscript, this careful summary, and their helpful suggestions. We have addressed the major and minor comments below.

Major Comments:

1. The pairwise correlation analysis revealed a significant marginal signal of known gene interactions. However, the amount of variation and large overlap between correlations for pairs with unknown relationships versus known is likely to mean that the use for developing hypotheses is minimal. That being said, there are published approaches for analysis of expression co-evolution that could be adapted to tissue bias, alternatives to Pearson's correlation that might prove to have better properties, and network context that may be relevant for evaluating observed correlations. For example, it is possible that noise and confounding signals (due to complexity of tissue samples) may vary in impact for different pathways, this could be evaluated and might allow for some filtering of informative/non-informative relationships. Partial correlation analysis might be used to explore issues of spurious correlation. There is also a lot of room for the development of an entirely novel approach. Ultimately, experimental validation may be needed to show the relevance of these relationships to identifying candidates with enough efficiency to be a feasible method of identifying good candidates.

RESPONSE: We appreciate the reviewers concern about the potential usefulness of these correlations in generating hypotheses, especially given the size of the matrix and the large range in correlations we report. We have taken their advice and incorporated an additional analysis more closely following existing publications, specifically the procedure for removing outliers described in Clark et al, 2012: https://genome.cshlp.org/content/22/4/714.full.pdf. The methods of that paper state:

"We were not interested in correlations driven by a single outlier data point, so we set a protein-specific limit of evolutionary rate at two standard deviations from the mean. This limit biased high correlations to those consistently involving multiple branches instead of one outlier branch."

We have applied a modified version of this filtering to our own analyses (l. 291-295, 613-615). In addition to the full correlation results we provided before, we now provide a matrix calculated only using transcripts with scaled changes within two standard deviations of the mean. Essentially, we filter out any transcripts for which we have reconstructed an extreme change in expression, and then calculate the correlations between the remaining transcripts. We provide these results in our data visualization and code repository as a more conservative set of correlations that could be queried for future hypothesis generation.

In response to their additional recommendations, we have opted to not develop a wholly novel approach, or to attempt to experimentally validate the correlations we have detected in this study, as we feel that falls outside the scope of this manuscript. But we are enthusiastic about seeing both of these avenues pursued in future publications.

In general, we feel that the data visualization we have created could prove useful to readers for filtering "good" candidates from this large set of correlations. This visualization allows researchers to immediately query their gene of interest, evaluate both the underlying data and the calculated correlations, and make an informed decision which putative interactions, if any, might be valuable to test.

2. It is challenging to biologically interpret changes in tissue bias for homology groups. Is it possible to use data from other species groups (where you could create a transcriptome with similar challenges – but have a better handle on ground truth) to better understand what happens in different cases (e.g. a paralog has increased expression in the ovary)? How will the expression estimates behave, more generally, when the number of member transcripts changes along a lineage?

RESPONSE: We agree that it can be challenging to conceptualize how variation within and across homology groups might impact results. To that end, we have included the data visualization at https://shchurch.shinyapps.io/hawaiian_fly_dataviz_2021/ to allow readers to explore and see expression values across transcripts for themselves. Using this, a reader can see [1] the number of transcripts in a group, [2] the variation across transcripts within a group, [3] the differences in average expression reconstructed on a phylogeny, and [4] the correlation between expression changes across groups.

We also hope that the same kinds of studies will be performed on different datasets, including those with robust, ground truth genomic references. However, we feel that incorporating such analyses on other species' expression data is out of the scope of this manuscript, which aims to document ovary expression evolution in Hawaiian Drosophila*.*

3. I recommend taking a second look at the first paragraph of the introduction and, manuscript-wide, the approach to citation. I know it is not deliberate, but the overall takeaway was an impression of devaluing the existing literature - rather than showing the novel aspects and advantages of your data set and analytical approach as intended. Similarly, in the discussion contextualization of the findings in relation to the existing literature is mostly 'big picture', and that made it difficult to understand the overall contribution being made. For example, how do the findings fit into what has been found in relavent vertabrate studies?

RESPONSE: We have taken the reviewer's thoughtful suggestion and have updated the language in our introduction to try and remove the emphasis from devaluing the literature, and instead point to what we feel our manuscript can offer to the field (l. 53, etc). In addition, we have included a summary statement to the discussion connecting our results to the recent study by Breschi et al in mammals (l. 334-336):

"In summary, consistent with recent findings in mammals (Breschi et al, 2016), our study shows that our expectation about the interspecific similarity of tissues should decrease as the phylogenetic distance increases, and the rate of that decrease will depend on the tissue in question."

Minor comments.

1. It seems like there might be a need to use assembly and/or mapping approaches designed to be robust with respect to sequence variability present among wild-caught individuals. Was this explored, and were specific approaches used to handle the sequence variability?

RESPONSE: We appreciate the concern shared by this reviewer and reviewer #3 about potential sources of variation across individuals. In this study, we account for variation in transcript counting efficiency across individuals (e.g. due to sequence variability) by performing analyses on tissue bias, calculated as the ratio of expression across treatments. We have included a clarification in the text (l. 124-126, 571-573):

"Using ratios is advantageous because any potential variation in transcript counting efficiency across individuals is canceled out, as this will be present in both the numerator and denominator"

2. Ovary-specific vs. ovary-bias – this should be clearly defined if the intent is that specific means something different from bias; otherwise, use bias throughout.

RESPONSE: We added the following clarification to the introduction (l. 121-124):

"Throughout the text we use ovary-specific and head-specific to refer to transcripts that are significantly differentially upregulated in the ovary or head relative to the carcass. We use the term ovary-biased and head-biased to refer to transcripts that have a positive ratio of expression between the ovary or head and carcass, regardless of magnitude."

3. I probably missed something, but I wasn't completely clear on the significance of the specific crossover point. Is it just a way to get a quantitative readout of whether an apples-to-apples comparison exists for tissue bias across a given phylogenetic distance?

RESPONSE: Yes that is what we intend to convey, the cutoff represents a point at which we expect more variation across species than across tissues. We include the following statement in the text to clarify (l. 327-330):

"[We identify] the crossover point at which variation between species begins to swamp variation across the tissues or treatments in question. In such comparisons, the possibility that any individual gene may show a divergent pattern of expression from the model organism increases substantially."

4. The behavior of which source of variance, tissue/species, predominates at a particular phylogenetic distance is likely to be affected by different degrees of complexity of the samples. You might expect the species variation to exceed 'tissue' at smaller distances when averaging across cell types/tissues in the more complex samples.

RESPONSE: We agree with the reviewer's conclusions, and we observe and report this phenomenon in our study in Figure S14 of our manuscript. Because we only test two tissues,

the ovary and the head, we refrain from drawing any conclusions about whether the difference we observe in time-to-crossover represents a global pattern, negatively correlated with tissue complexity, or a singular incident.

5. Pg 16 lines 261-264: With respect to assigning changes to ovary or head versus to the carcass, what was the overall landscape in terms of changes in ovary/head alone and changes in both? Was filtering done based on this?

RESPONSE: We treat the ovary vs. carcass and head vs. carcass comparisons completely independently, and perform no analysis or filtering on conditions using both. A comparison of the number of up- and down-regulated genes between the ovary and head is included in supplemental figures S5 and S6 and in the text of the results.

6. Pg 17 lines 279-281: As mentioned by the authors, 'head' is a more complex set of organs, tissues, and cell types. It seems likely that this would reduce the signal for co-evolutionary changes in expression between interacting genes.

RESPONSE: We appreciate the reviewer's suggestion for how to interpret the results in Figure S20. In that figure we show that, when considering the head-carcass comparison, there is not the same significant difference in expression correlation between physically interacting genes and genes with no or known interactions. As mentioned above, since we only test two tissues, the ovary and the head, we refrain from drawing any conclusions about whether this discrepancy is due to tissue complexity or some other factor. We state in our manuscript that "the strength of this signal may be dependent on the tissues being compared" (l. 306)

7. Pg 19 lines 848-850: Do the homology groups reduce statistical noise or are they used to reduce systematic error that might result from artifacts produced by the assembly?

RESPONSE: We replaced the phrase "statistical noise" with "variation due to assembly artifacts" (l. 373). Averaging over homology groups allows us to reduce variation due to fragmentation during assembly.

8. Pg 19 852-853: What is the expectation here and how can we evaluate the strength of the concordance? Is the concordance driven by single orthologs or low complexity homology groups?

*RESPONSE: We appreciate the reviewer's note and have removed the qualifier "*strong*" from that sentence, as we cannot evaluate the strength of concordance without better null expectations. With this paragraph, we hope to emphasize that we observe concordance in expression patterns across evolutionary time and in relation to what has been reported for* D. melanogaster, *and this adds to our confidence in the robustness of our approach.*

9. How issues of multiple testing are handled is not addressed clearly in the methods.

RESPONSE: We have included text in the methods communicating that p-values for differential gene expression were adjusted using the Benjamini and Hochberg correction, which is the default in DESeq2 (l. 513)

We double checked that the adjusted p-values, and not the raw p-values, were used in reporting significance throughout the manuscript. In section 3.1 we found several statements that were incorrectly based on raw p-values. These have been updated to use the adjusted p-values, for example (l. 155-157)

"When comparing the head to the carcass, we observed an average of 7% *(previously 10%)* of transcripts to be significantly upregulated and 7.2% (*previously 10.5%)* to be significantly downregulated (Fig. S6)."

Reviewer #3: In this manuscript Church et al. explore the evolution of ovary biased gene expression in Hawaiian Drosophila using RNA-Seq to estimate gene expression levels, and comparative methods to identify biases in gene expression by tissue and determine how these may have evolved. I find this to be a very nice study, both in the question asked and the methods used, that those interested in gene expression evolution will find of interest. I have only two concerns, which I don't think require additional experiments or data analyses (at least for point 1) but which would be useful to have a discussion about:

RESPONSE: We thank the reviewer for their helpful comments and kind remarks.

1) Clarification on how replicates were used (if this is stated I missed it, so might need to be more explicit if already in the text). Specifically, the methods state that 4 replicates were used, which is great, except for a single species, which is understandable. But it is not clear to me if gene expression estimates across replicates were kept as individual estimates (for results described in 3.1) or were then averaged for results described in 3.2, 3.3, and 3.4. If the results were averaged for results in 3.2, 3.3, and 3.4 it would be helpful (probably essential) to know what effects averaging might have on the results. For example, the average gene expression value can be the same for genes with very different variances in expression so how does variance in gene expression affect the results, such as the probability that gene gains or losses bias? Are genes with large variance in expression across individuals more likely to change bias or the inverse, or is there no relation?

RESPONSE: We have clarified in our description of results that (l. 212-213):

"in both datasets (based on homology groups or strict orthology groups), we averaged expression values per species across biological replicate individuals."

We appreciate the point the reviewer raises about variation across individuals, and how this may impact our conclusions. However, we face a challenge in this study, and in nearly every differential gene expression study, in that we are typically limited to very few samples across

species (here usually 3, and sometimes as low as 2). We acknowledge that, with such low sample sizes, we cannot hope to accurately calculate the variance of expression across individuals. In this study we have several such sources of variance that we do not control for (e.g. age and life-history across wild caught individuals); we acknowledge these openly in the text and point to our multiple sensitivity tests as evidence of the robustness of our results to these sources of variation.

2) Why reconstruct bias, rather than ancestral gene expression levels from which you re-estimate bias? Again, this relates to the variance in gene expression. From what I understand, the bias estimates are point estimates of single value and do not incorporate information on variation in gene expression levels within species (ie., between individuals). But this seems important, so why not reconstruct gene expression levels for all individuals and species, then estimate bias for individuals and get information on them mean and variance in bias estimates?

RESPONSE: We have added the following statement to the manuscript to clarify why we use bias, rather than absolute expression values (l. 571-573):

“Using ratios is advantageous because any potential variation in transcript counting efficiency across individuals is canceled out, as this will be present in both the numerator and denominator”

To answer the reviewers question, we reconstruct bias to [1] answer questions about the evolution of relative expression, rather than absolute expression, and [2] to account for potential variation across individuals. For example, as mentioned above, there may be sources of variation specific to an individual’s genome, or other factors. By analyzing expression bias, we account for these factors by focusing on the relative rather than absolute measure of expression.

We calculate bias within each individual, and then calculate the average bias across species and use this in our analysis. As mentioned above, we appreciate the reviewer’s concern about variance across individuals, and share their concerns about the limitations of findings in differential gene expression studies with low sample sizes and therefore a reduced understanding of gene expression variation. We point to the robustness of our results to multiple sensitivity tests, as well as our emphasis on sharing the raw data openly, as interim solutions until more data can be brought to bear on the problem.

3) A minor point, but how can gene expression changes in branches 21 and 13 be inferred, doesn't the method use an unrooted tree?

RESPONSE: We apologize for the oversight and have added the graphical representation of the root to the tree in Figure 4. We use a rooted tree for all analyses.
