# Supplementary material for: The evolution of ovary-biased gene expression in Hawaiian Drosophila
Source: PLoS Genet. 2023 Jan 23;19(1):e1010607. doi: 10.1371/journal.pgen.1010607 (PMC9894553; doi:10.1371/journal.pgen.1010607)
Supplement: Attachment — Submitted filename: 20221215_ReviewerResponse2_HawaiianDrosophila_PLOSGenetics.pdf [file pgen.1010607.s003.pdf]

December 15, 2022

Dear Dr. Kopp and Dr. Bomblies,

We thank the reviewers for their constructive reviews of our submitted manuscript. We have addressed all of the updated concerns, as detailed in our point-by point response below. We hope that you and the reviewers will find the revised manuscript suitable for publication.

Our responses to all verbatim points raised by reviewers are provided below in blue text. Changes to the manuscript made in response to these reviews are indicated in red text.

We look forward to hearing from you.

Sincerely,

Samuel H. Church

Major Comments:

• The added filtering seems reasonable, does it change the result in terms of the relationships shown in Figure 5? Is there a reason for choosing no to report the outcome of this alternative approach in the manuscript itself? I was not able to evaluate if this had a significant impact or not.

RESPONSE: Removing the outliers does not change the relationships shown in figure 5A and B, and we have added text to the manuscript that states this (line 312-314). The correlation networks shown for the example gene family in 5C, D, and E are changed from the removal of the gene families with low-confidence outliers (260 of these gene families have been filtered out). We point the reader to the visualization to see the networks estimated on both datasets (line 323-325).

Minor Comments:

• Adding an explanation of when/if to implement the filtering option might be helpful for users of the data visualization tool.

RESPONSE: We have added the following text to the data visualization:

“The dataset can be filtered to remove low-confidence outliers (260 gene families with estimated changes in expression greater than two standard deviations from the mean). Removing these outliers results in more conservative network estimates, but also means that some gene families will have no resulting relationships to display.”

• Using the option seems to result in some genes/families not visible (adgf is an example), and I didn’t find cases where it seemed to make a difference – but I also just spot checked. I am not sure if this is the expected behavior.

RESPONSE: This is the expected behavior, as the filtering step removes 260 gene families that have a change in expression beyond two standard deviations from the mean change. We have included the following language in the visualization to explain why some genes disappear:

“Removing these outliers results in more conservative network estimates, but also means that some gene families will have no resulting relationships to display.”
